# Human Error: The Impact of Job Insecurity on Attention-Related Cognitive Errors and Error Detection

**DOI:** 10.3390/ijerph16132427

**Published:** 2019-07-08

**Authors:** Lara Christina Roll, Oi-ling Siu, Simon Y.W. Li, Hans De Witte

**Affiliations:** 1 Department of Applied Psychology, Lingnan University, Hong Kong, China; 2 Optentia Research Focus Area, North-West University, Vanderbijlpark 1900, South Africa; 3 Work, Organisational, and Personnel Psychology Research Group, KU Leuven, 3000 Leuven, Belgium

**Keywords:** quantitative job insecurity, qualitative job insecurity, error detection, behavioral data, cognitive errors, burnout

## Abstract

(1) Background: Work-related stress is a major contributor to human error. One significant workplace stressor is job insecurity, which has been linked to an increased likelihood of experiencing burnout. This, in turn, might affect human error, specifically attention-related cognitive errors (ARCES) and the ability to detect errors. ARCES can be costly for organizations and pose a safety risk. Equally detrimental effects can be caused by failure to detect errors before they can cause harm. (2) Methods: We gathered self-report and behavioral data from 148 employees working in educational, financial and medical sectors in China. We designed and piloted an error detection task in which employees had to compare fictitious customer orders to deliveries of an online shop. We tested for indirect effects using the PROCESS macro with bootstrapping (3) Results: Our findings confirmed indirect effects of job insecurity on both ARCES and the ability to detect errors via burnout. (4) Conclusions: The present research shows that job insecurity influences making and detecting errors through its relationship with burnout. These findings suggest that job insecurity could increase the likelihood for human error with potential implications for employees’ safety and the safety of others.

## 1. Introduction 

In the era of the gig economy, global trade wars and uncertain political environments, such as Brexit (the United Kingdom leaving the European Union) [1], job insecurity is on the rise [2]. Scientific interest in the concept of job insecurity started with Greenhalgh and Rosenblatt’s [3] authoritative article, ‘Job insecurity: Toward Conceptual Clarity’. Since then, extensive research has documented the negative consequences of job insecurity on employees’ well-being and health [4,5]. The concept of job insecurity implies uncertainty about the future. It is therefore different from actual dismissal. Employees who are certain that they are going to lose their jobs can prepare for the future, while employees faced with job insecurity do not know what to expect. Thus, the concept of job insecurity has an underlying involuntary nature, producing a discrepancy between what employees wish for (i.e., certainty about the future of their employment) and what they get (i.e., uncertainty about their job future) [6]. This discrepancy does not only include people’s fear of losing their job versus stable employment (quantitative job insecurity), it also includes the discrepancy between individuals’ valued job aspects, like a short commute to work, and the potential prospect of losing them (qualitative job insecurity). In general, job insecurity puts employees in a situation in which their job future and all resources connected to it are uncontrollable and unpredictable [4].

Job insecurity has been widely described as a workplace stressor [5,7]. If this stress becomes chronic, it can manifest itself as severe exhaustion, commonly referred to as burnout [8]. Symptoms of burnout have been linked to reduced performance, job satisfaction and the ability to process information [9,10,11]. Specifically, researchers have related the level of burnout symptoms to attention lapses [11]. Attention lapses are human and part of everyday life [12]. However, while some of those errors are merely inconvenient, such as forgetting to pick up an item at the grocery store, others can result in serious injuries or even deaths, impacting personal as well as organizational safety [13]. 

Apart from burnout, job insecurity has also been identified as a potential risk factor to safety outcomes in the workplace [12,13]. Employees reporting high perceptions of job insecurity have shown decreased safety motivation and compliance. This may be due to less cognitive resources being available when employees are preoccupied with the future of their jobs [11]. Therefore, in the present research, we aim to examine the following research question: Is job insecurity related to an increased number of cognitive errors among employees, and is burnout an underlying factor of this relationship? To address this question, we have conducted a study in which we include both self-reports and behavioral data in the form of a newly designed error detection task.

### 1.1. Quantitative and Qualitative Job Insecurity

Job insecurity has been defined as a “concern about the continued existence of a current job” [14]. It is important to highlight that job insecurity is a subjective perception [15]. The same objective situation can be interpreted differently depending on employees’ perception. On the one hand, despite an objectively insecure situation, employees might feel confident they will retain their jobs. On the other hand, employees might be highly concerned about the future of their employment without an objective reason. Researchers have noted that the perception of a situation is a stronger predictor for outcomes than the objective situation [16], which is why we focus on employees’ perceived level of job insecurity in this study. It should, however, be noted that in general individuals’ subjective perception of job insecurity corresponds well with the objective probability of job [15].

The impact of this subjective experience also extends beyond the objective reality of employees’ job contracts. Research has shown that both permanent and temporary employees experience job insecurity [5]. Contrary to the intuitive belief, studies have shown that the association between job insecurity and negative consequences is much more pronounced for employees on permanent than temporary contracts. As a potential explanation, psychological contract breach has been proposed [17]. Permanent employees expect from their employers job security in exchange for their loyal work. Thus, when faced with job insecurity, they perceive a breach of their psychological contract leading to negative outcomes. Temporary workers, on the other hand, do not have the same expectation from their employers and hence the impact of job insecurity might be less severe.

The literature has distinguished two types of job insecurity: Quantitative and qualitative [18]. Quantitative job insecurity refers to whether employees feel they will be able to keep their jobs or might become unemployed. In contrast, qualitative job insecurity is concerned with being insecure about valued job characteristics, e.g., wage, the location of employment or working hours [15]. The question that has been evoked was which type of job insecurity is more problematic. Research findings on the different influences of quantitative and qualitative job insecurity have been mixed [5]. Hellgren et al. [18] found quantitative job insecurity to predict health and well-being, while qualitative job insecurity predicted job satisfaction and turnover intention. Roskies and Louis-Guerin [19] found a stronger relationship for qualitative job insecurity and job satisfaction than for quantitative job insecurity and job satisfaction. To further investigate the difference between quantitative and qualitative job insecurity, Handaja and De Witte [20] used a more differentiated measure and found results supporting Roskies and Louis-Guerin’s [19] findings. In a major research effort, De Witte et al. [21] undertook a study to compare the two different types of job insecurity to a wide range of outcomes. Results did not show clear differences between the influences of quantitative and qualitative job insecurity. The authors concluded that both types seem to be problematic for health and well-being. Given these equivocal results, the present research aims to further investigate the influences of quantitative and qualitative job insecurity and examines both types in the present study and whether they have different effects on everyday cognitive errors.

### 1.2. Cognitive Errors

Failures in everyday life can be distinguished into mistakes and slips [22,23]. Mistakes occur if people have incorrect or insufficient knowledge of the task they are attempting to perform. For example, if a doctor fails to make the correct diagnosis due to lack of knowledge of a specific illness. Slips, in contrast, occur despite people having the correct knowledge. Instead, people take the wrong action in completing a task. For example, in haste the doctor mixes up the files of two patients, resulting in the wrong diagnoses being entered into the patients’ database. In the latter example the doctor has the required knowledge to make a correct diagnosis yet slips up, resulting in a false record. Slip errors are difficult to avoid and can even happen to highly skilled experts. In the present research, our focus is on slip errors, to which we refer as attention-related cognitive errors (ARCES) [24]. For example, an everyday task for many people is to drive to the local grocery store. After a while, most people know how to get there without requiring directions. If someone still took a wrong turn, it would be an example of a cognitive error. In this scenario, not much harm would be done. However, people who report high-frequency ARCES, tend to be more likely to cause automobile accidents [25]. Thus, ARCES can have very serious safety consequences for individuals themselves and people around them. The link between perceptions of job insecurity and safety is attracting a growing body of research [13,26]. In one of the first studies on this relationship, Probst and Brubaker [12] showed in a longitudinal study that job insecurity was associated with low safety motivation and compliance, which is related to higher accident and injury rates at the workplace. Since then, numerous other studies have confirmed this link [27,28,29,30].

As a theoretical explanation for the negative consequences of job insecurity, conservation of resources (COR) theory has been proposed [31]. According to COR theory, people strive to minimize resource loss. Secure work is one of those resources, especially since it is essential to obtain other resources like housing, electricity, food, social support and more. For this reason, job insecurity is experienced as stressful [32]. In turn, experiencing this stress results in the decline of other resources [33], such as the cognitive resources required to avoid ARCES.

Apart from COR theory, threat rigidity theory (TRT) provides a theoretical framework for the influence of job insecurity on cognitive errors [34]. In TRT, a threat leads people to behave with more rigidity [35]. As a consequence, their focus shifts to dominant cues only and less information is processed overall. Thus, people’s ability to focus attention and concentrate is impaired, making them more prone to cognitive errors [11].

Everyday cognitive errors have the potential to impact employees’ safety, as well as the safety of everyone working around them. In addition to quantitative job insecurity, we also examine the threat of losing valued job characteristics in the form of qualitative job insecurity in this study. Since previous research has demonstrated similar negative effects for both [21], we hypothesize the following:

**Hypothesis** **1:** Both quantitative and qualitative job insecurity will be significantly positively related to ARCES.

### 1.3. Indirect Effects via Burnout 

The stress associated with job insecurity poses a threat for individuals to ‘burn out’ [36]. In the literature, burnout is characterized by cynicism (i.e., a cynical and negative attitude towards one’s work), emotional exhaustion (i.e., drained emotional resources) and lack of professional efficacy (i.e., reduced belief to be able to fulfill one’s own professional role) [37]. Individuals suffering from burnout tend to have less mental and physical energy available, making them more prone to cognitive errors, accident and injuries [38]. For example, Shanafelt et al. [39] reported that burnout was strongly related to medical errors and Nahrgang et al. [40] found a relationship to reduced safety behavior in the workplace.

In the present study, we expect that when individuals experience job insecurity, they are more likely to experience burnout, which in turn makes them more prone to cognitive failures. We base these proposed relationships on the job-demands resources (JD–R) model [41]. This model proposes a dual process theory [42]: Job strain on the one hand and work motivation on the other hand are affected by two different underlying psychological processes: One process is motivational and assumes that job resources relate to work engagement, which results in higher work motivation. The other process, which is the relevant process for our research, assumes that job demands trigger a health impairment process. Job demands exhaust employees’ physical and mental resources, making them more prone to burnout and subsequently ill health as well as lower performance. Job insecurity has commonly been classified as a job demand that can trigger this health impairment process [43]. Thus, according to the JD–R theory, job insecurity drains employees of their mental and physical resources, opening them up for burnout, which impairs their performance and possibly mental ability to concentrate. Therefore, drawing on the JD-R model, we hypothesize the following:

**Hypothesis** **2:** Burnout will have a positive indirect effect on the relationship between both quantitative and qualitative job insecurity and ARCES.

### 1.4. Error Detection: Misses and False Positives

Despite the error-prone nature of our cognitive systems, humans survive and thrive [44]. In fact, the reason that we function well despite erring is that we can detect errors and correct them, ideally before they are causing harm. When an error is made, detecting it helps humans to adapt behavior and avoid further errors in the future [45].

Cognitive errors can be divided into (a) missing an error and (b) falsely identifying an error. Reason [46] referred to these two different types as (a) errors of omission and (b) errors of commission. Errors of omission occur when an individual fails to recognize a problem and does not try to solve it, e.g., a poor-quality item gets accepted. Other researchers refer to errors of omission as misses [47]. In contrast, errors of commission refer to improper actions, i.e., correcting an error where there is none. For example, a good quality item gets rejected. Errors of commission are also commonly referred to as false positives [48]. For the present research, we adopt the terminology of misses and false positives to refer to these two different types of cognitive errors.

Our ability to detect errors is a very important component of our cognitive control [49]. For example, accuracy in error detection is vital in radiology and security screenings, as missed tumors or contraband might have life-threatening consequences [50]. The consequences of errors might range from mild annoyances to huge personal or even global disaster. Therefore, in the present research, we do not only examine cognitive errors employees make (ARCES), but also the number of errors that go unnoticed (misses) and are falsely identified (false positives). For this purpose, we implement a newly designed error detection task to identify how many errors employees fail to spot or identify incorrectly. Drawing on the COR [31] and TRT theory [34], we hypothesize that employees’ level of job insecurity will impact their cognitive functioning, which in turn will affect their ability to detect errors:

**Hypothesis** **3:** Both quantitative and qualitative job insecurity will be significantly positively related to misses.

**Hypothesis** **4:** Both quantitative and qualitative job insecurity will be significantly positively related to false positives.

Failure to detect errors might occur due to (a) not recognizing a problem, (b) overlapping responsibilities or diffusing responsibilities of individuals and (c) work overload, stress and burnout [38,51]. Applying the health impairment process from the JD–R theory [43], we expect that both quantitative and qualitative job insecurity will pose job demands, creating a higher risk for burnout, which will affect employees’ performance on the error detection task:

**Hypothesis** **5:** Burnout will have a positive indirect effect on the relationship between both quantitative and qualitative job insecurity and misses.

**Hypothesis** **6:** Burnout will have an indirect effect on the relationship between both quantitative and qualitative job insecurity and false positives.

### 1.5. The Present Research 

This study was conducted with a Chinese sample from state-owned facilities (banks, schools and hospitals). These employees represent the more traditional Chinese workforce that used to enjoy lifelong tenure [47]. Nowadays, Chinese governmental policies have changed, and employees of state-owned facilities can be dismissed. However, they still tend to experience higher levels of security in their jobs than employees of other organizations in China, like joint ventures [48].

A strength of this study is that we included a behavioral measure as an outcome variable in addition to self-reports, which reduces the common-method bias. Specifically, we designed a task in which participants had to detect errors. In research, visual search tasks are a popular method to assess error detection [52]. This is because visual searches are highly relevant in everyday life, e.g., when searching for a friend in a crowded place or for a specific item on a shelf. Yet, to the best of our knowledge, this is the first attempt of a study to link job insecurity to both self-reported ARCES and behavioral error detection.

## 2. Materials and Methods 

### 2.1. Pilot Studies

Since we designed an error detection task for this research, we piloted it two times before conducting the actual study. The objective of the pilots was to determine the length of the study and construct validity of the newly developed error detection task. First, we piloted the English version of the error detection task with a convenience sample of six students from Lingnan University (Hong Kong) and subsequently revised it. Second, after translating the task into Chinese (Mandarin), we piloted the whole study with employees, in this case 10 staff members working at the canteen at Lingnan University (Hong Kong). Based on insights from the pilots, the error detection task and overall study procedures were refined and finalized. For example, we determined that in order to keep the overall duration of the study to a maximum of one hour, we could include two practice and 12 actual study trials for the error detection task. Furthermore, based on the results from the two pilots, we determined that one minute and 45 seconds was the ideal amount of time to allow for each trial. This was enough time for most participants to comfortably complete a trial while maintaining momentum and moving forward with the study.

### 2.2. Participants and Procedure 

This research was carried out with employees from banks, hospitals and schools (all state-owned facilities) in Shanghai (China) during June and July 2014. The majority of participants came from schools, though the exact numbers were not recorded. The researchers obtained gatekeeper approval from employees’ supervisors to conduct the study after working hours with volunteering participants. The study was conducted on-site in large rooms with several employees at the same time, though great care was taken to ensure that employees would not work together, e.g., by seating participants as far away from one another as possible and reminding participants to work individually.

In total, we had 148 participants, of which 63% were female. The average age was 42.3 years with a standard deviation (SD) of 9.3 years, ranging from 23 to 61 years. Most participants were married (91.6%). Almost all of them were employed full-time (98.6%) and the average tenure was 20.3 years (SD = 10.3 years). About two-thirds had a permanent employment contract (66.9%) and participants worked an average of 38.9 hours per week (SD = 15.0 hours/week).

In terms of education, the Chinese education system is divided into elementary (six years of schooling until ages 11 or 12), lower secondary (nine years of schooling until ages 14 or 15) and higher secondary (12 years of schooling until ages 17 or 18), followed by university [53]. The majority of participants indicated that their level of education was higher secondary (78.8%). Only 0.7% indicated they had no formal education, 1.5% reported they had elementary education, 11.7% indicated they had lower secondary education and 7.3% had attended university.

The procedure of this study followed three steps: In the first step, participants were told that the study required them to verify information. Participants were further informed that their participation was voluntary, and that anonymity would be ensured. All subjects gave their informed consent before participation in this study. The study was conducted in accordance with the Declaration of Helsinki, and ethical approval had been obtained following the guidelines for human ethics approval as outlined by the Research Grants Council (RGC), Hong Kong. In the second step, participants were provided with the error detection task. They completed two practice trials before moving on with the actual trials. All participants were informed that both accuracy and speed are important, but that they should not sacrifice accuracy for speed. The researchers acted as timekeepers and informed participants when to move on to the next trial, even if they were not finished with the previous trial yet. In the third step, participants were asked to fill in a questionnaire. Afterward participants were debriefed, thanked for their participation and received RMB80 (equivalent to about USD12) as a token of appreciation for their time.

### 2.3. Measures

#### 2.3.1. Questionnaire

Since the questionnaire was administered in Chinese (Mandarin), all scales were back-and-forth translated following the procedures outlined by Brislin [54]. Internal reliabilities of individual scales are displayed in Table 1. We included the following scales in this study:

*Quantitative job insecurity* was measured with De Witte’s [36] job insecurity scale, which has been validated by Vander Elst, De Witte, and De Cuyper [55]. Participants were asked to rate the items on a six-point Likert scale, ranging from 1 (Strongly disagree) to 6 (Strongly agree). The scale showed a Cronbach’s alpha of 0.62, which was below the recommended cut-off point of 0.70 [56]. Thus, we inspected Cronbach’s alphas with individual items deleted from the scale. Results showed that the reliability of the fourth item (“I am sure I can keep my job.”) was low. One possible explanation is that while three items in this scale ask participants to indicate the extent to which they feel insecure (e.g., “I feel insecure about the future of my job.”), the fact that the fourth item was reversed may have been overlooked by several participants. For this reason, we decided to drop the fourth item, which resulted in a Cronbach’s alpha of 0.77 for the three-item scale.

*Qualitative job insecurity* was examined with four items tapping into aspects described by De Witte et al. [21]. An example item is, “I feel insecure about the characteristics and conditions of my job in the future.” Answer options were the same as for the quantitative job insecurity scale. The four-item scale had a low Cronbach’s alpha of 0.57. Inspecting the reliability coefficients when specific items were dropped, a three-item scale showed best reliability at 0.68 (excluding the item “Chances are, my job will change in a negative way.”). Since this value is close enough to the cut-off of 0.70 to suggest satisfactory reliability (e.g., see [57]), we retained the three-item scale for all further analyses.

*Burnout* was assessed with the 16-item Maslach Burnout Inventory-General Survey [58]. The scale measured exhaustion (e.g., “I feel ‘burned out’ by my work”), cynicism (e.g., “I became more cynical about the effects of my work”) and professional efficacy (e.g., “I achieved a lot of valuable things in this job”; reversed-coded). The six-point Likert scale ranged from 0 (Never) to 6 (Always). Cronbach’s alpha was acceptable at 0.70. The license for this tool was purchased from Mind Garden (http://www.mindgarden.com).

*Attention-related cognitive errors* were measured with the 12-item scale by Cheyne, Carriere and Smilek [24], e.g., “When reading I find that I have read several paragraphs without being able to recall what I read.” Answers were given on a six-point Likert scale ranging from 0 (Never) to 6 (Always). Cronbach’s alpha was high at 0.90. 

*Demographic* measures included participants’ age, gender (0 = female, 1 = male), level of education (1 = no formal qualification, 2 = lowest formal qualification, 3 = above lowest formal qualification, 4 = higher secondary qualification, 5 = university degree), relationship status (0 = single, 1 = married/cohabitating/living with family or parents), tenure in years, employment status (0 = full-time, 1 = part-time), number of working hours per week, contract type (0 = permanent, 1 = temporary) and level of English proficiency (“How many years have you been studying English?”).

#### 2.3.2. Error Detection Task

Error detection was assessed by applying a specifically designed error detection task. In this task, two types of errors could occur [38]: (a) Misses (undetected or omission errors) and (b) false positives (commission errors), i.e., anything incorrectly identified as an error when it was in fact none. The error checking task consisted of checking orders from a fictitious online shop and was designed to resemble real-life online orders. The task was paper-based and consisted of two practice and 12 actual trials (based on the pilots). For each trial, participants were presented with three sheets of paper: (1) The customer order, (2) the billing receipt and (3) the checksheet (see Appendix A Figure A1, Figure A2, Figure A3, Figure A4, Figure A5, Figure A6, Figure A7, Figure A8, Figure A9, Figure A10, Figure A11, Figure A12, Figure A13, Figure A14, Figure A15, Figure A16, Figure A17, Figure A18, Figure A19, Figure A20, Figure A21, Figure A22, Figure A23, Figure A24, Figure A25, Figure A26, Figure A27, Figure A28, Figure A29, Figure A30, Figure A31, Figure A32, Figure A33, Figure A34, Figure A35, Figure A36, Figure A37, Figure A38, Figure A39, Figure A40, Figure A41 and Figure A42 for all material used in the error detection task). The checksheet was the same for each trial and listed 10 categories the participants were supposed to check.

To prevent potential order effects, trials were pseudo-randomized with the constraint that no more than two consecutive trials contained an error. The rationale was to prevent all trials containing errors to randomly appear in the beginning or end of the error detection task by chance. Participants were randomly allocated to those pseudo-randomized trials.

To prevent potential confusion regarding which customer orders and billing receipts should be compared and to further prevent any potential mix-up of checksheets, corresponding papers were marked by an animal symbol in the right top corner of each page. For example, corresponding customer order, billing receipt and checksheet would all three have a rabbit symbol in the top right corner. Animal symbols were chosen, instead of for example numbers or letters, to prevent participants from making assumptions about the order of trials. Moreover, if we had used numbers or letters, participants might have been confused about why trials did not follow chronological orders after the pseudo-randomization.

**Figure A43:**
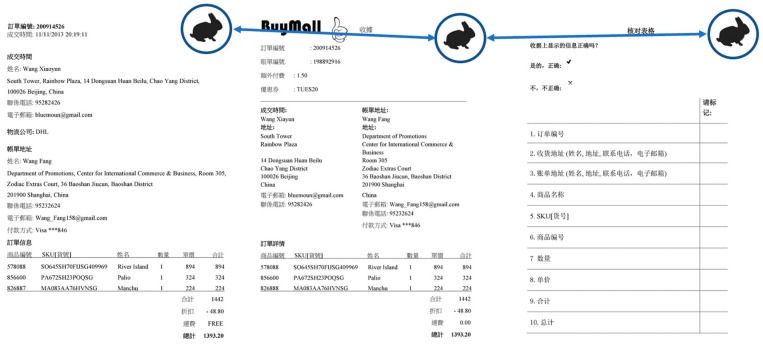
This figure shows the customer order (**left**), which needed to be compared to the billing receipt (**middle**). Results needed to be marked on the checksheet (**right**). The animal symbol identified which papers needed to be compared.

**Figure A44:**
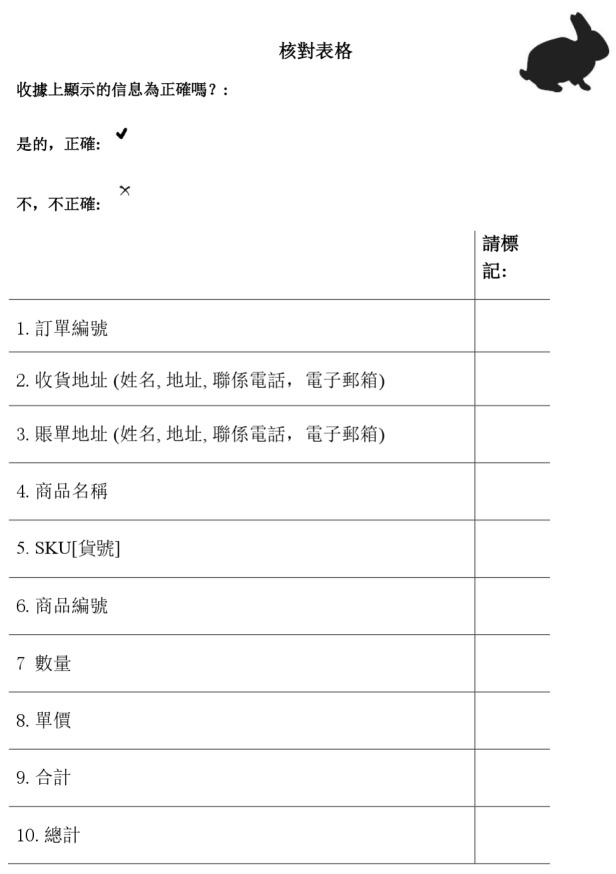
The checksheet listing 10 items to be compared.

**Figure A45:**
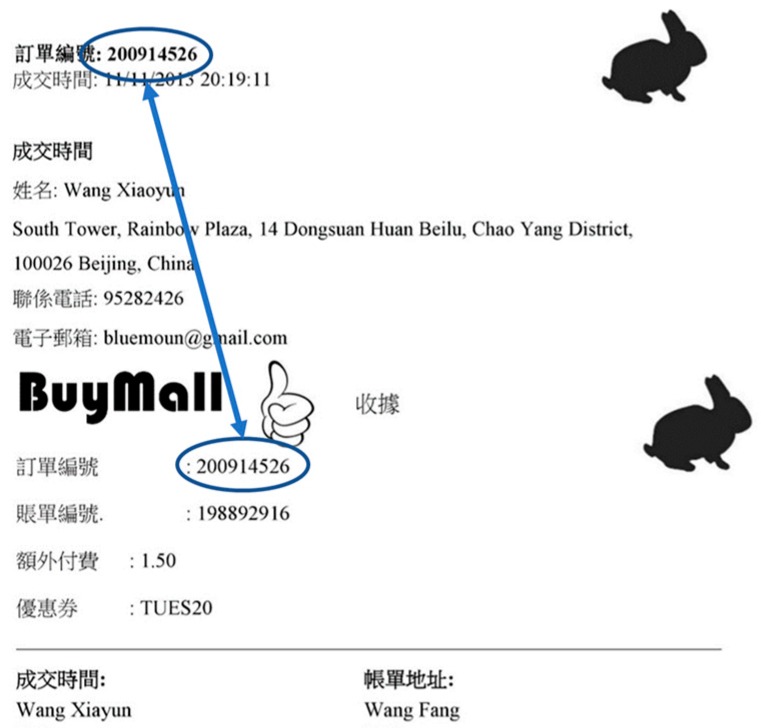
Example of the first item on the checksheet that needed to be compared: order number.

**Figure A46:**
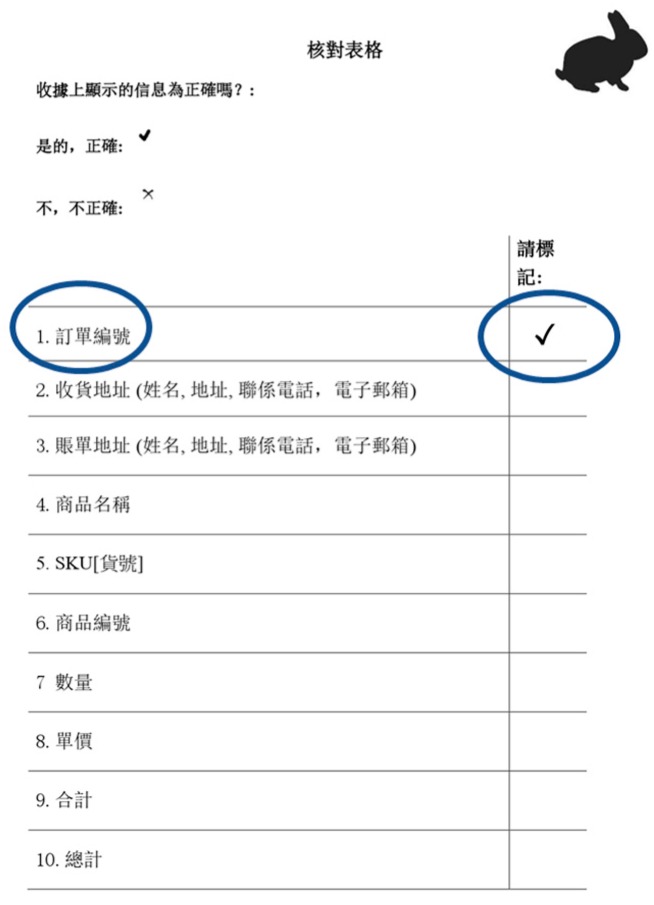
Participant marking that the first item was correct on the checksheet.

**Figure A47:**
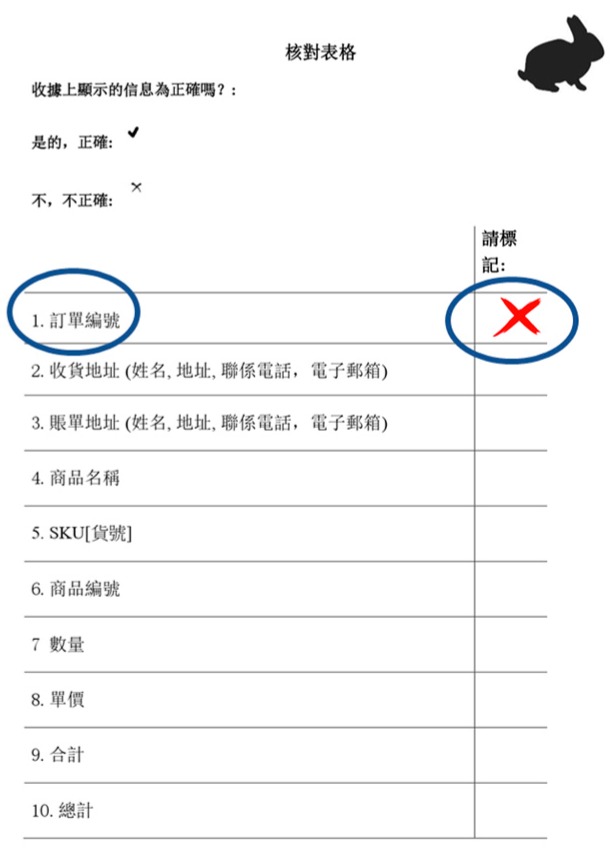
Participant marking that the first item was incorrect on the checksheet.

Participants needed to check the order against the billing receipt and indicate the result on the checksheet (see Appendix B Figure A43, Figure A44, Figure A45, Figure A46 and Figure A47 for an illustration of the procedure). For example, one item on the checksheet required participants to check the order number. Thus, participants first had to locate the order number on the customer order and on the billing receipt. Second, they had to compare whether the order number was the same on both sheets. If it was, they had to put a tick behind the item “order number” on the checksheet. If it was not the same, i.e., it was incorrect, they had to put an “X” behind “order number” on the checksheet.

#### 2.3.3. Principles Underlying the Error Detection Task

The error detection task was developed in English and then back-and-forth-translated [54]. All information on the customer order, billing receipt and checksheet not containing errors were translated into Chinese simplified characters (Mandarin). The errors were contained in the numbers on those sheets and the customer addresses as well as product names. The reason why the customer addresses and product names were not translated into Chinese characters were two-fold: One, exchanging a whole Chinese character as an “error” in a trial appeared too easy for Chinese native speakers to spot. Two, when people order goods online, they can enter their addresses in pinyin, the romanization of Chinese characters based on their pronunciation. Thus, people tend to be familiar with the pinyin spelling of addresses and use it in everyday life. Therefore, we kept the addresses written in romanization.

**Figure 1:**
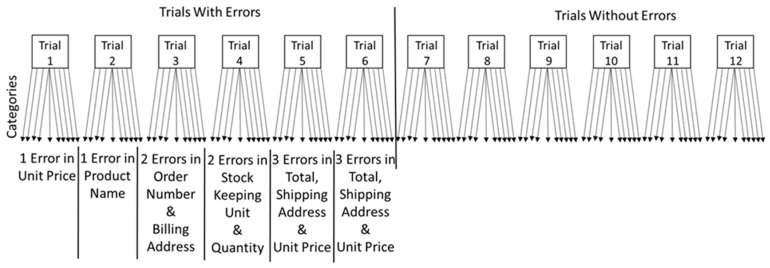
Overview of trials and respective errors.

In every trial, there were 10 categories participants needed to check (see Figure 1). Wolfe [59] and Wolfe, Horowitz and Kenner [60] reported that in visual screening tasks the typical error (“target”) rate is 50%. Hence, in the present study, the likelihood of an order to contain an error was held constant at a rate of 50%, meaning six out of the 12 trials contained errors. Based on previous research, we set the error rate within those categories at 20%, meaning there was a total of 10 errors in the experiment [38]. The rationale for holding error probability constant was to increase the likelihood that a significant effect was due to different levels of job insecurity and burnout.

The types of errors were based on the categorization by Wiseman, Cairns and Cox [61]. The researchers developed a framework to classify errors. For the present study, we focused on three error types (see Table 2). The first error type was labeled “digit(s) added”, e.g., 5.06 for 5.6. The second error type was “incorrect pattern use” and referred to cases in which the original numbers were mixed up, but no new numbers were added, e.g., 1464 for 1646. The third type of error was “out by one” and meant that the number was one larger or smaller than the original, e.g., 83.81 for 82.81. Those error categories were initially developed for numbers only. For the purpose of this experiment, we transferred the error categories to errors with letters. For example, an error of the category “incorrect pattern use” was a misspelled word or name, in which two letters would have been switched, e.g., “Kunming Road” instead of “Kumning Road”.

Previous research has shown that error or target prevalence (frequency or rarity by which an error/target occurs) influences the detection rate [60]. Specifically, Wolfe et al. [60] studied “misses” (failures to notice a target) in an artificial baggage-screening task. They discovered that target rarity leads to inaccurate performance. People were significantly less likely to detect a rare target as compared to a frequent target. To rule out target prevalence as a potential confounding variable, we kept the target prevalence constant. Given that the error detection task contains 10 errors, two error types (“digits added”; “out by one”) occur three times, while the third type (“incorrect pattern”) occurs four times, respectively.

### 2.4. Analytical Strategy 

We performed our analyses in SPSS 25 software (IBM, Armonk, NY, USA), First, we extracted means, standard deviations (SDs), correlations and to investigate the reliability of the constructs calculating Cronbach’s alpha coefficients. To test the hypothesized direct and indirect effects, we used model 4 (5000 bootstrapping resampling) in the PROCESS macro developed by Hayes [62]. As pointed out by Hayes, Montoya and Rockwood [63], separate regressions in the analysis of indirect effects is advisable over the use of a structural equation modeling (SEM) approach with small sample sizes. This is due to the default estimation methods used by most SEM programs relying on large sample asymptotic theory. Hence, maximum likelihood standard errors tend to be biased downward in small samples [64]. 

In the present analysis, bootstrapping does not make assumptions about normal distribution. In this procedure, 5000 resamples are drawn from the data, each time calculating the direct and indirect effects. For an indirect effect to be significant, the 95% confidence interval (CI) must not include zero. In order to determine which variables to control for, we examined correlations between our dependent variables (i.e., ARCES, misses and false positives) and demographic variables. Education, age, gender, tenure, weekly working hours and contract type were significantly correlated with either ARCES, misses or both. Thus, we included those six variables as covariates in the analysis.

#### Analytical Strategy for the Error Detection Task

Misses (i.e., undetected errors) and false positives (i.e., the detection of errors that were none) were counted for each participant and percentages of those misses and false positives were calculated. For misses, it was determined whether the participant checked every trial. Trials or items not checked were identified by the absence of a response in the form of a cross or tick on the checksheet. 5.1% of participants did not check every trial containing an error. For those participants, the percentages of misses were based on the actual number of checked errors: The maximum number of possible misses in the task was 10 (i.e., the task contained 10 errors in total). If a participant only checked trials containing seven out of the 10 errors in total, their rate of misses was based on seven errors as 100%. If participants detected all errors, their rate of misses was 0%. For every missed error, the error rate increased respectively.

The rate of false positives was calculated in percentages for each participant based on the actual number of checked categories in each trial within the given time frame. This rationale was based on the theory of Wiseman [61]. Theoretically, the number of possible false positives is infinite. However, in this particular task, the maximum number of false positives could be identified as the maximum number of possibilities for identifying a false positive based on the number of categories checked. There were 12 trials with a total of 120 categories. 10 categories contained errors, meaning there were 110 possibilities for false positives.

## 3. Results

Means, standard deviations and correlations are reported in Table 1. As expected, job insecurity was positively related to burnout (quantitative job insecurity: *r* = 0.29, *p* < 0.01; qualitative job insecurity: *r* = 0.36, *p* < 0.01). Neither qualitative nor quantitative job insecurity was significantly correlated with any of the outcome variables (i.e., ARCES, misses and false positives). However, several researchers have argued that even if there is no significant direct correlation between an independent variable and an outcome, there might still be a significant indirect effect through a third variable [65,66], as we have hypothesized in the present study.

Burnout was significantly positively correlated with ARCES (*r* = 0.59, *p* < 0.01) and misses (*r* = 0.16, *p* < 0.05). Previous research found very low rates of false positives from which no conclusions could be drawn [34,42]. Similarly, in this study the rates of false positives were very low and not significantly correlated with any other study variables. Therefore, we rejected H4 and H6 and excluded false positives from further analyses.

### 3.1. Validity of the Error Detection Task

**Figure 2:**
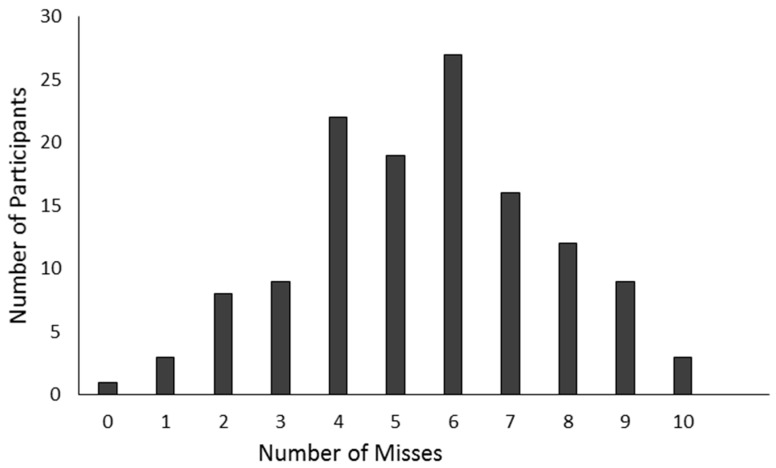
Error detection task: Distribution of misses across participants.

Figure 2 shows the absolute number of errors missed by the number of participants in the error detection task. For example, 22 participants out of the total of 148 participants missed four out of 10 errors in total. As can be seen from this figure, the graph is close to a normal distribution. If the task had been too easy, the graph would have been skewed and shown that most participants had detected the majority of errors. If the task had been too difficult, the majority of participants would not have been able to detect most errors. Therefore, it can be assumed that the error detection task was neither too easy nor too difficult. Thus, the error detection task appeared to be valid. Another indication of task validity is that misses significantly correlated with self-reported ARCES (*r* = 0.19, *p* < 0.05). 

### 3.2. Direct and Indirect Effects

In H1 we hypothesized a direct effect between both quantitative and qualitative job insecurity and ARCES. Bootstrapping results in PROCESS indicate that neither direct effect was significant (quantitative job insecurity: *B* = −0.02, *SE* = 0.06, *t*(120) = −0.03, *p* > 0.05; qualitative job insecurity: *B* = −0.08, *SE* = 0.07, *t*(120) = −1.14, *p* > 0.05), which means that H1 was rejected. In H2 we hypothesized an indirect effect of job insecurity on ARCES via burnout. Results confirmed H2 for both quantitative (*B* = 0.13, *SE* = 0.41, 95% CI (0.06, 0.22)) and qualitative job insecurity (*B* = 0.20, *SE* = 0.05, 95% CI (0.11, 0.30)).

For H3 we hypothesized that there would be a direct relationship between both quantitative and qualitative job insecurity and misses. Similar to the results above, neither direct effect was significant (quantitative job insecurity: *B* = −1.13, *SE* = 3.03, *t*(117) = −2.75, *p* > 0.05; qualitative job insecurity: *B* = −1.27, *SE* = 1.98, *t*(117) = −0.64, *p* > 0.05). Thus, we have rejected H3. Lastly, in H5 we expected an indirect effect of job insecurity on misses via burnout, which could be confirmed for both quantitative (*B* = 1.33, *SE* = 0.64, 95% CI (0.19, 2.66)) and qualitative job insecurity (*B* = 1.93, *SE* = 0.92, 95% CI (0.29, 3.91)).

## 4. Discussion 

Preventing cognitive errors as well as detecting errors before they can cause harm is vital for organizational safety as well as for the safety of others, like patients in a hospital or passengers on a plane. This study aimed to investigate relationships between job insecurity and self-reported cognitive errors on the one hand and behavioral error detection on the other hand. In order to combat common method bias, we have developed an error detection task as a behavioral performance outcome.

Findings supported that job insecurity impacted both making and detecting errors, and that these relationships occur through burnout. This provides empirical support for burnout as an essential underlying factor the occurrence of ARCES and misses when employees experience job insecurity. Furthermore, the present study provides support that not only fear of losing the job as a whole (i.e., quantitative job insecurity) but also the fear of losing valued job characteristics like wage or location (i.e., qualitative job insecurity) can be risk factors for employees to make more errors and to be less likely to detect errors before they can cause harm to themselves or others.

ARCES were strongly correlated with burnout. This supports our theory and previous findings that burnout and cognitive errors are closely linked [11]. We found similarly strong relationships between burnout and ARCES in a separate study, in both a different Chinese and German sample [67], providing further empirical evidence that this relationship appears to be consistently strong. 

In line with previous research, the present study did not find a significant effect for false positives in the error detection task [60]. It appears that false positives have very low occurrence rates in visual search tasks, making it difficult to draw meaningful conclusions. 

### 4.1. Theoretical Implications

To the best of our knowledge, this study is the first to describe empirical findings indicating that job insecurity as an antecedent to burnout can impact cognitive functioning. As such, the current study enhances knowledge about the potential behavioral manifestations of job insecurity. Moreover, our results provide further support for that people’s self-perception of ARCES reflects their actual cognitive performance [11]. Despite appearing intuitive, such associations have not always been found in related research. For example, patients of chronic fatigue syndrome often report concentration problems, yet perform as well as control groups on various cognitive tasks [68]. Researchers have proposed that these patients either misinterpret their cognitive performance abilities or hold themselves to unrealistic standards [69]. In contrast, findings from this study suggest that employees facing burnout due to job insecurity appear to have realistic insights into their cognitive performance. Lastly, our results contribute to the ongoing debate about the differential influences of quantitative and qualitative job insecurity [21]. Specifically, we provide further empirical support that both types are in fact equally problematic.

### 4.2. Practical Implications

With this study, we provide a new practical tool for future use in research on cognitive errors. In general, our findings indicate that job insecurity can affect employees’ cognitive abilities through burnout. Thus, it indicates that those employees may experience cognitive challenges at work when facing job insecurity, possibly further undermining their chances of retaining their job or valued job characteristics. Aiming to maintain sustained performance might even lead to more stress, creating a potential downward spiral. This cognitive impairment should be considered in the treatment of burnout and in the implementation of interventions at the workplace.

### 4.3. Limitations and Future Research

Since the error detection task was newly developed, more research is needed applying this tool to perfect and validate it. Though we have conducted two pilot studies to improve the task as much as possible before conducting this study, we believe that it will be even better applying the tool electronically in the future. As we were going into our participants’ workplaces and we did not have enough electronic devices at our disposal, we had to rely on a paper-and-pencil task. However, programming the task on a computer would carry the benefit of exact and automatic time-keeping, among others.

Though collecting behavioral data was a first step to combat cross-sectional data bias, taking further steps, like collecting longitudinal data, is advisable in the future. Moreover, since our sample was relatively small, we could not split it to conduct more in-depth analyses on differential impacts by industry, gender, age or other demographic variables. Collecting data from a larger sample and conducting these additional analyses will likely yield new and insightful findings.

Additional limitations of the second study included that conditions could not be manipulated, and participants could not be assigned randomly. Therefore, it is not a true experiment and no claim regarding causal relationships can be made. 

A number of recommendations can be made for future research. First, more studies are needed examining how human error detection can be improved, possibly applying true experimental and/or longitudinal research designs to disentangle the causal influences of job insecurity. Further, the search for buffers of the relationships between job insecurity and health/performance outcomes is highly important. One of the core tasks of researchers in this field is to develop clear interventions and empirically evaluate their effectiveness to reduce the detrimental effects of job insecurity [5].

## 5. Conclusions

Organizational change is not always avoidable and thus reducing job insecurity is not always possible, especially in turbulent economic times. Findings of this study suggest that if job insecurity is unavoidable, it is essential for organizations to seek effective strategies to help employees cope in order to avoid costly mistakes, accidents and injuries in the workplace [70].

## Figures and Tables

**Table 1:** Means, standard deviations, Cronbach’s alphas and correlation matrices (*N* = 148).

Variables	Means	SDs	1	2	3	4	5	6	7	8	9	10	11
1. Quant. JI	2.09	1.07											
2. Qual. JI	2.46	0.97	0.58 **										
3. BO Total	2.89	0.60	0.29 **	0.36 **									
4. ARCES	2.82	0.78	0.13	0.11	0.59 **								
5. Misses	54.28	22.05	0.10	0.03	0.16 *	0.19 *							
6. False Pos.	1.84	3.01	−0.01	−0.11	0.01	0.05	−0.11						
7. Edu	*n.a.*	*n.a.*	−0.08	−0.01	−0.02	−0.01	−0.27 **	0.09					
8. Age	42.3	9.3	0.13	0.04	0.03	0.11	0.43 **	−0.05	−0.11				
9. Gender	*n.a.*	*n.a.*	0.19 *	0.09	−0.09	−0.17 *	0.26 **	−0.01	−0.06	0.23 **			
10. Tenure	20.3	10.3	0.01	−0.02	0.03	0.18 *	0.31 **	−0.05	−0.05	0.90 **	0.13		
11. Hours/w	38.9	15.0	−0.09	−0.01	−0.03	−0.07	−0.28 **	−0.10	0.01	−0.25 **	−0.11	−0.17	
12. Contract	*n.a.*	*n.a.*	0.04	0.16	0.06	−0.03	−0.28 **	0.01	−0.05	−0.57 **	−0.02	−0.52 **	0.11

Note. Quant. JI = Quantitative Job Insecurity, Qual. JI = Qualitative Job Insecurity, BOEE = Burnout Emotional Exhaustion, BOCYN = Burnout Cynicism, BOPE = Burnout Professional Efficacy, BO Total = Burnout Total, ARCES = Attention-related cognitive errors, False Pos. = False Positives, Edu = Education, Hours/w = Hours worked per week, Contract = contract type (permanent vs. temporary), *n.a.* = not applicable. “Misses” and “False Positives” are indicated in percentages. * *p* < 0.05, ** *p* < 0.01.

**Table 2:** Error types of the error detection task.

Error Type	Example
1. Digit(s) wrong	826887 for 826878
2. Digit(s)/Letter added	Quyiang Road instead of Quyang Road
3. Incorrect pattern use	Kumning instead of Kunming
4. Out by one	2 instead of 3

Note. Based on Wiseman, Cairns and Cox, 2011.

**Figure A1:**
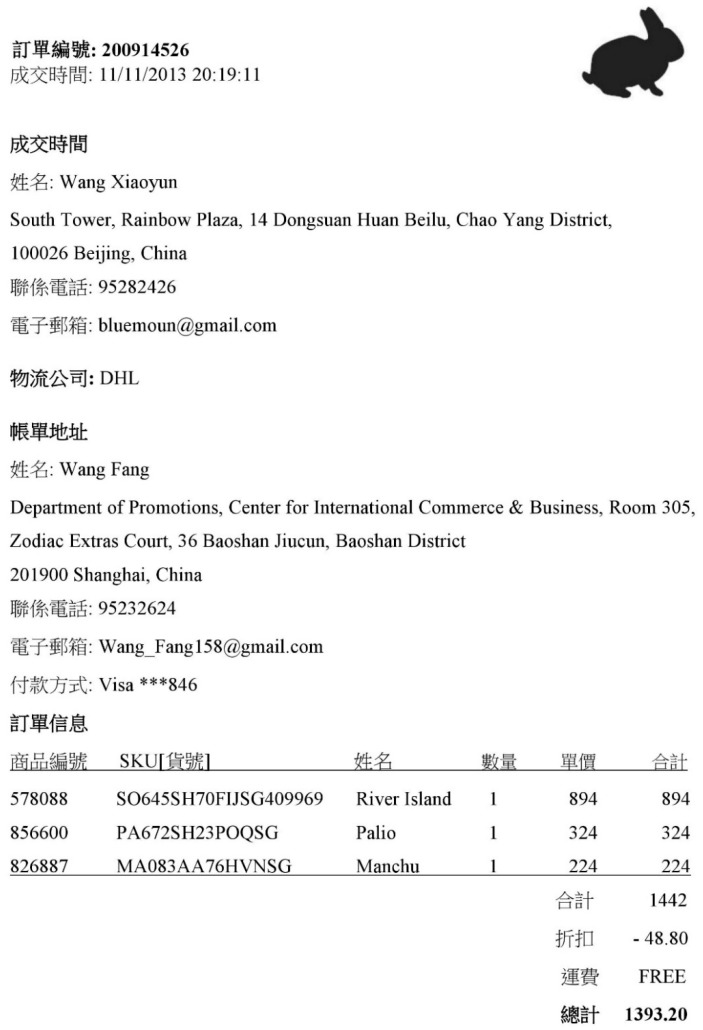
Practise trial rabbit: Customer order.

**Figure A2:**
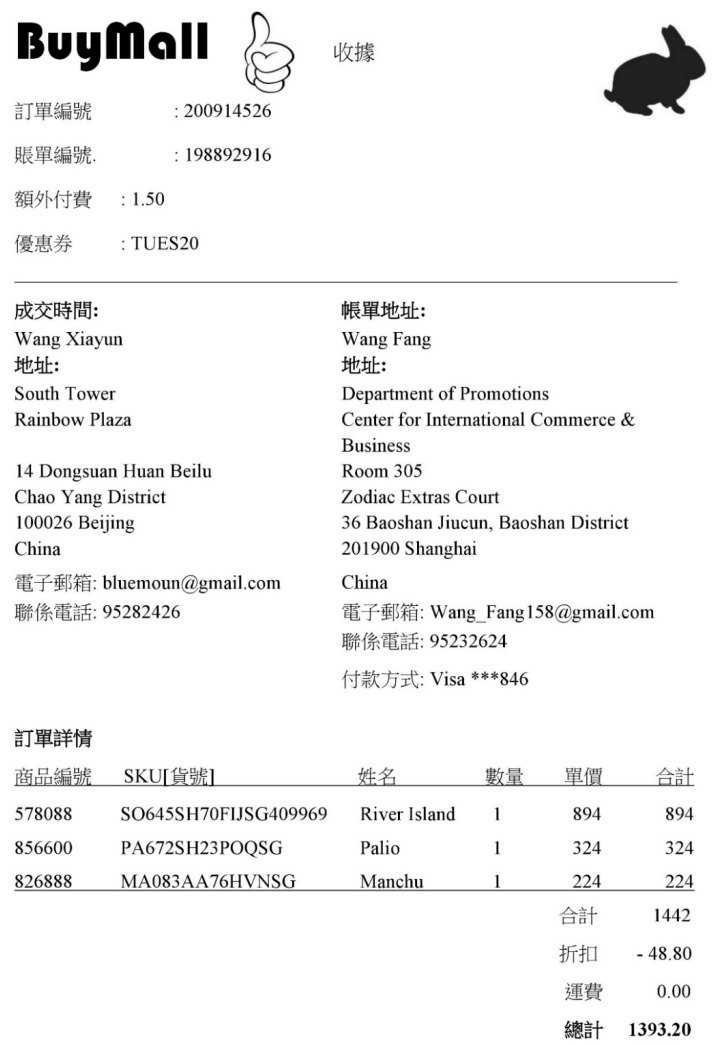
Practise trial rabbit: Billing receipt.

**Figure A3:**
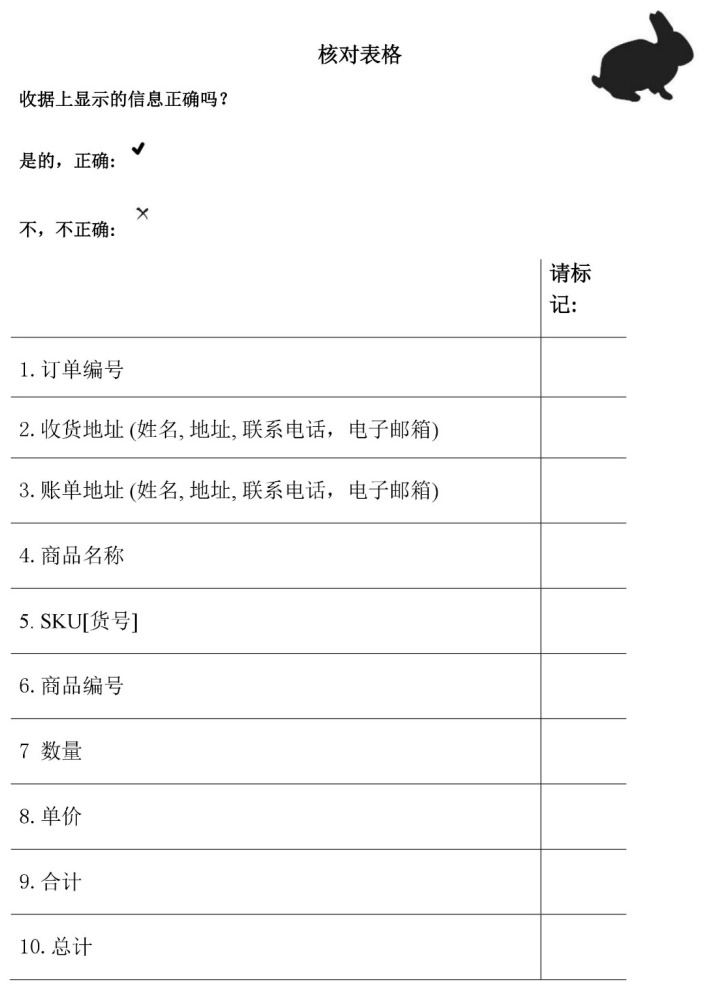
Practise trial rabbit: Checksheet.

**Figure A4:**
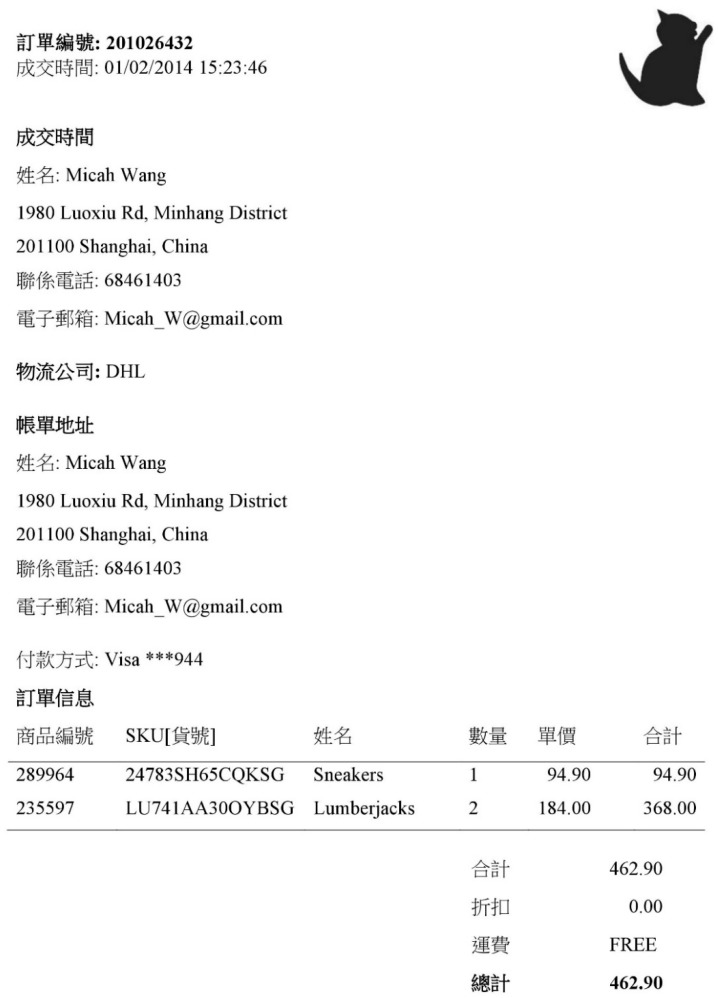
Practise trial cat: Customer order.

**Figure A5:**
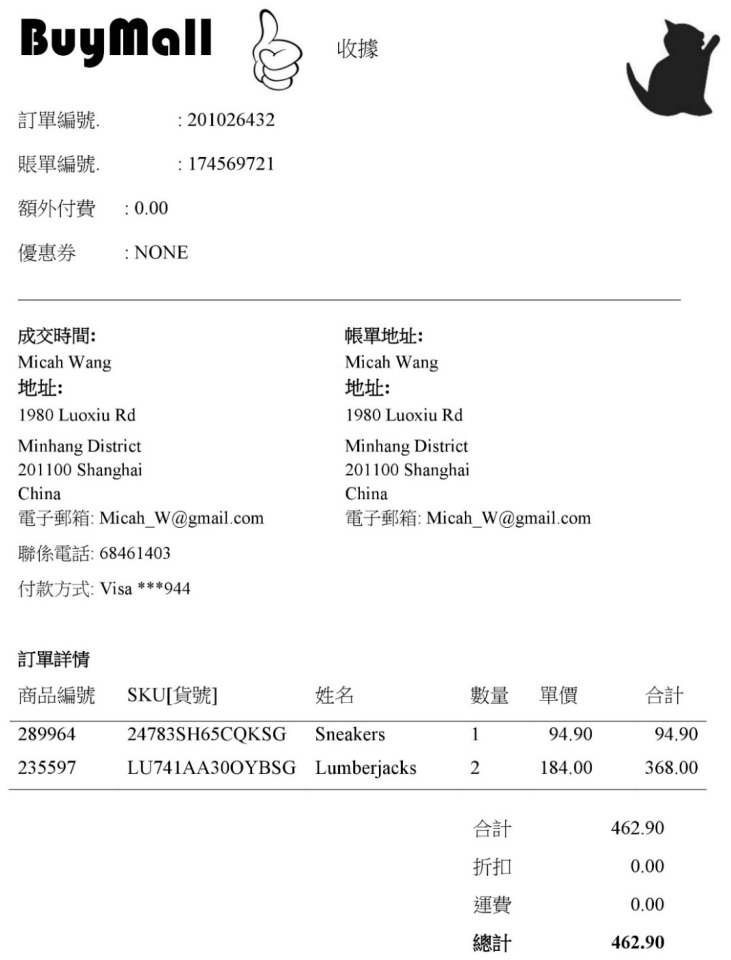
Practise trial cat: Billing receipt.

**Figure A6:**
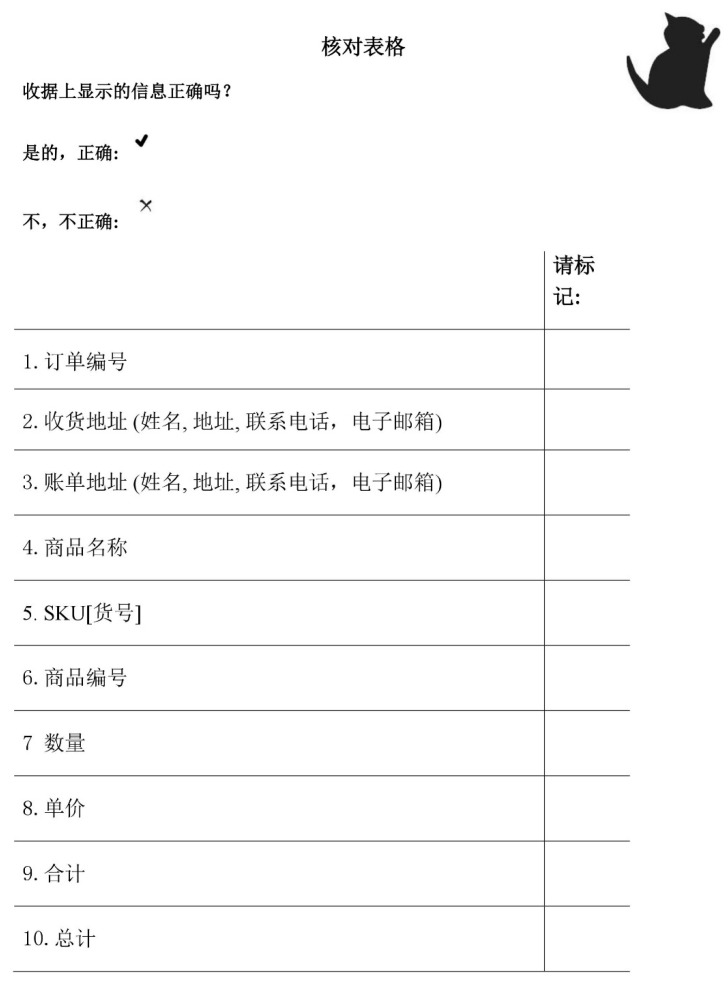
Practise trial cat: Checksheet.

**Figure A7:**
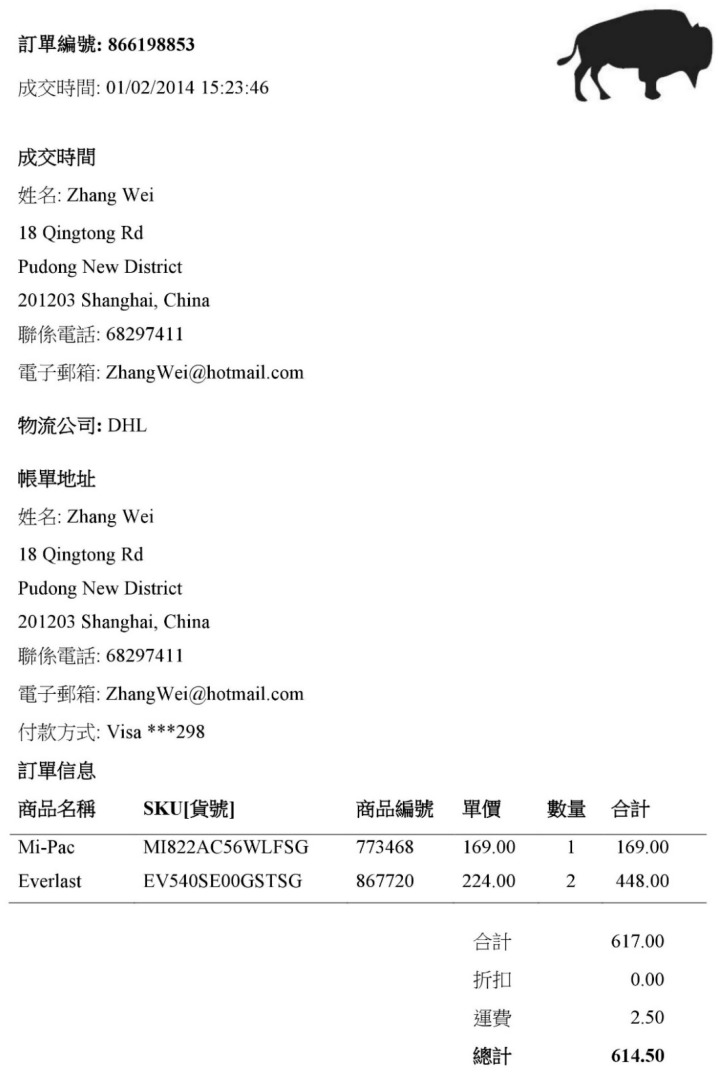
Study trial buffalo: Customer order.

**Figure A8:**
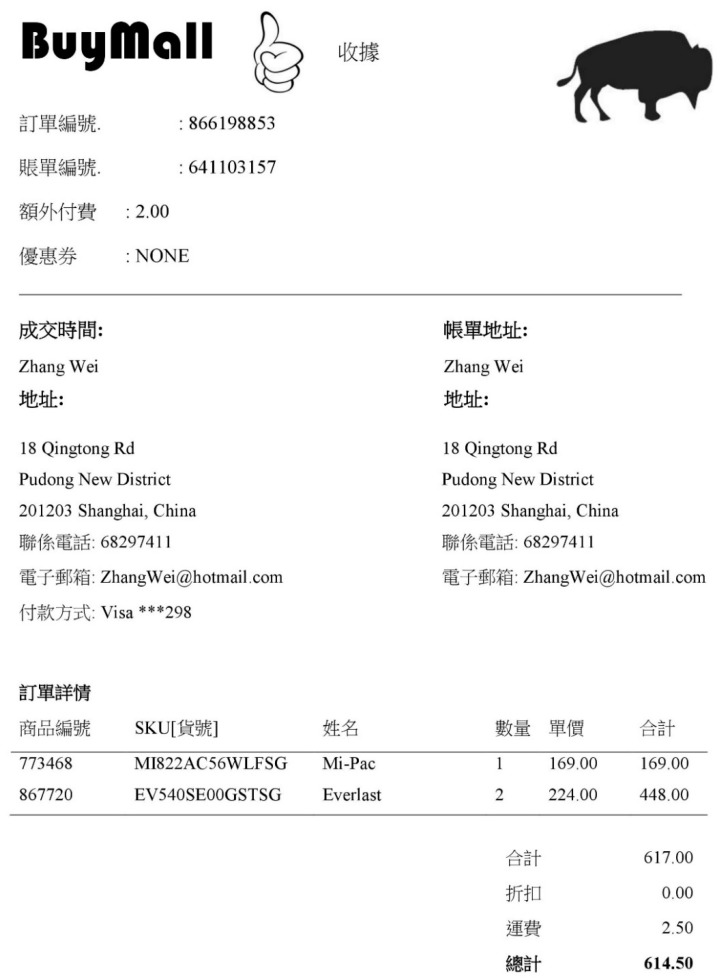
Study trial buffalo: Billing receipt.

**Figure A9:**
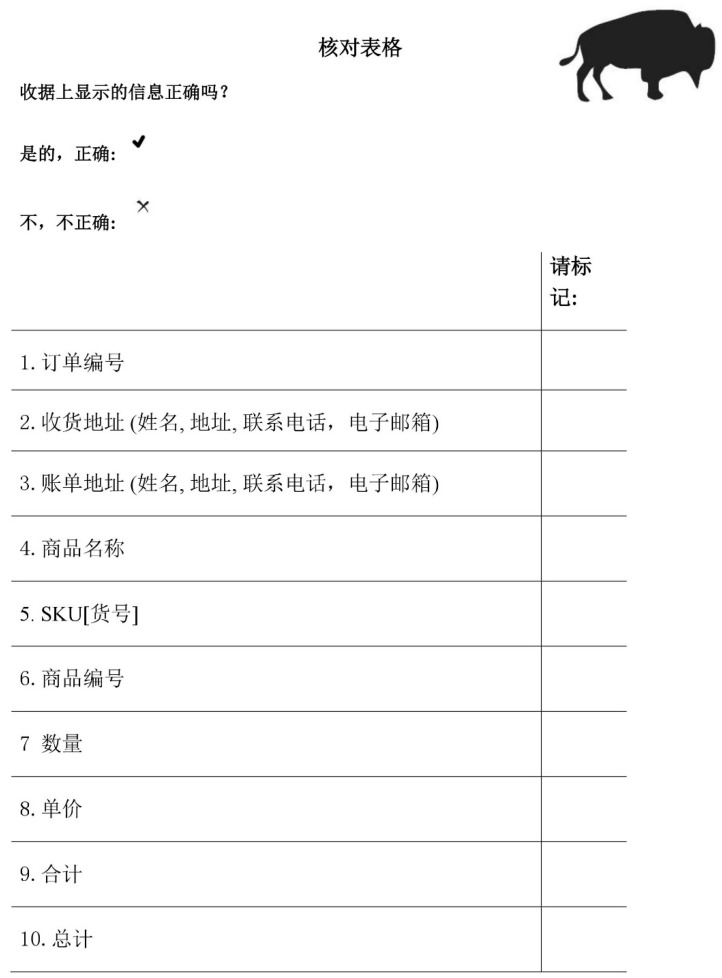
Study trial buffalo: Checksheet.

**Figure A10:**
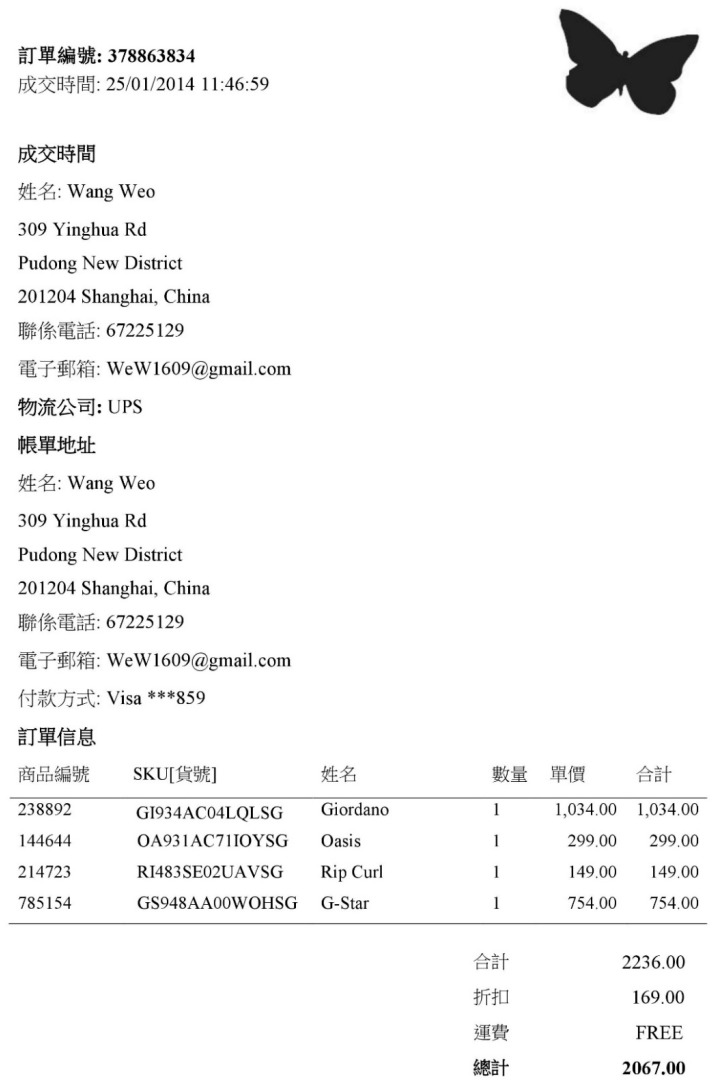
Study trial butterfly: Customer order.

**Figure A11:**
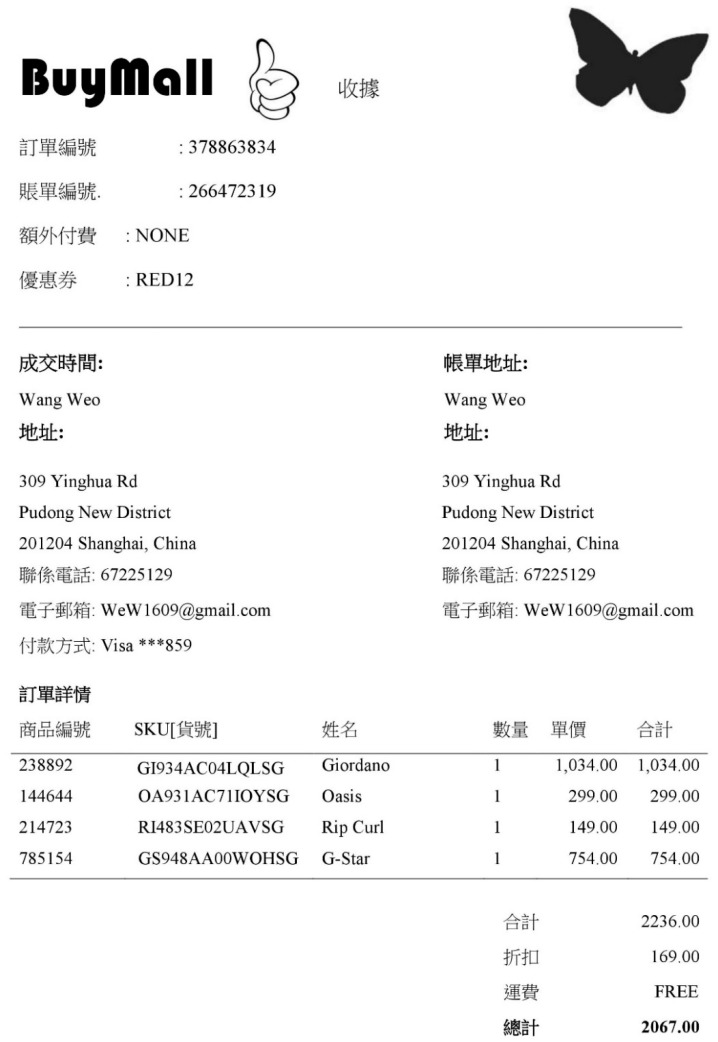
Study trial butterfly: Billing receipt.

**Figure A12:**
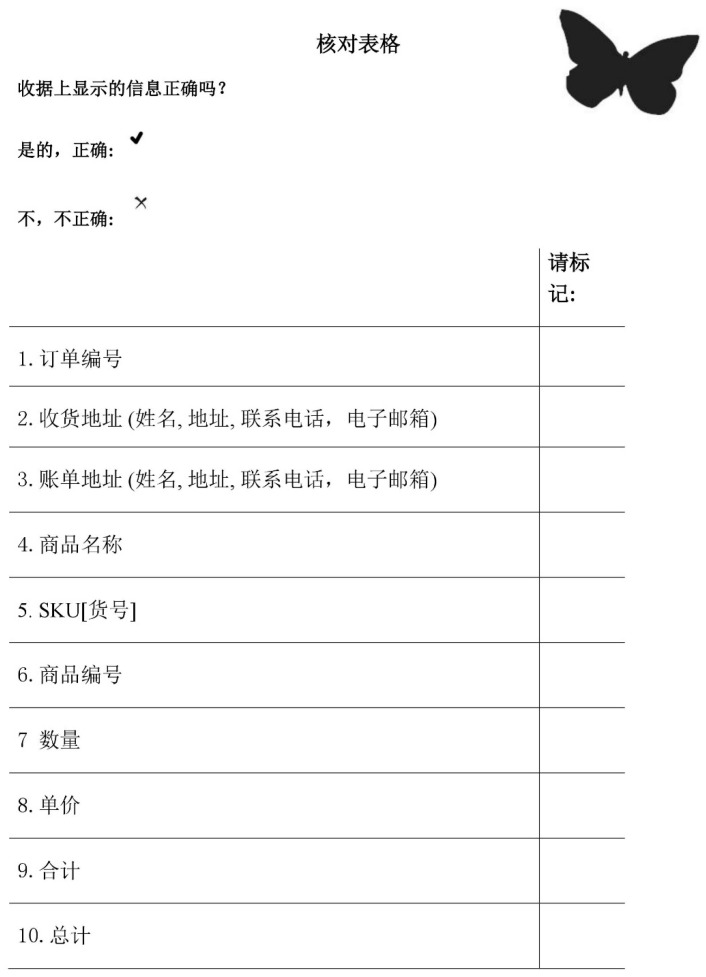
Study trial butterfly: Checksheet.

**Figure A13:**
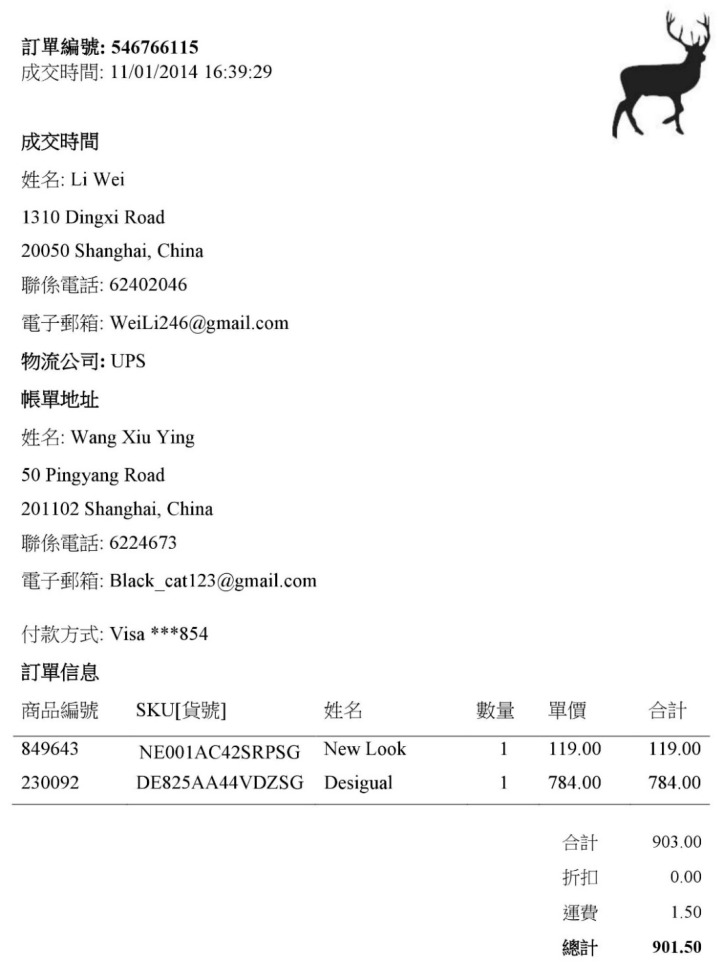
Study trial deer: Customer order.

**Figure A14:**
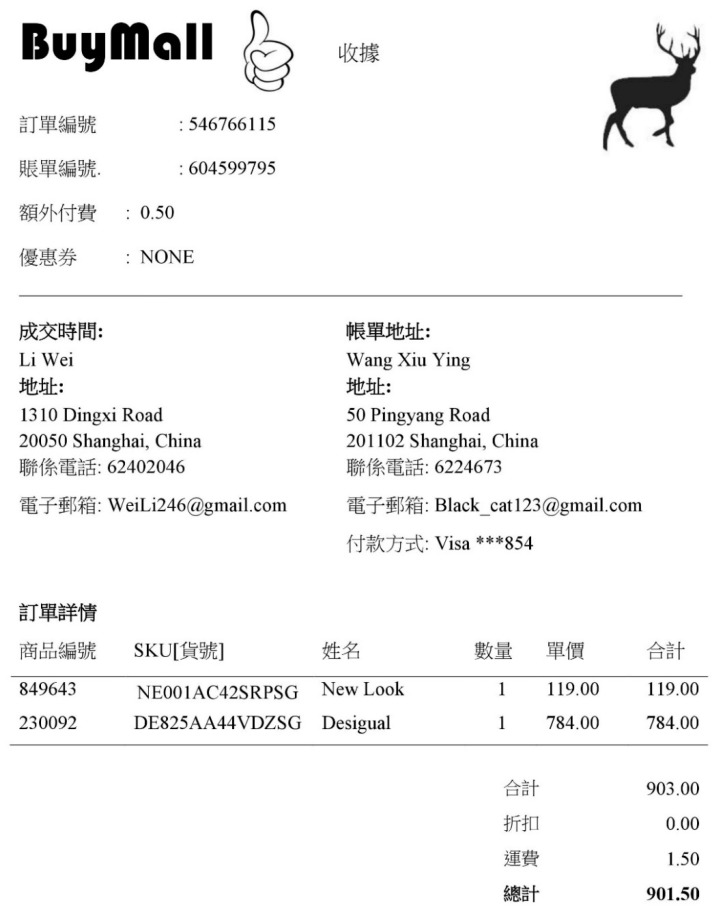
Study trial deer: Billing receipt.

**Figure A15:**
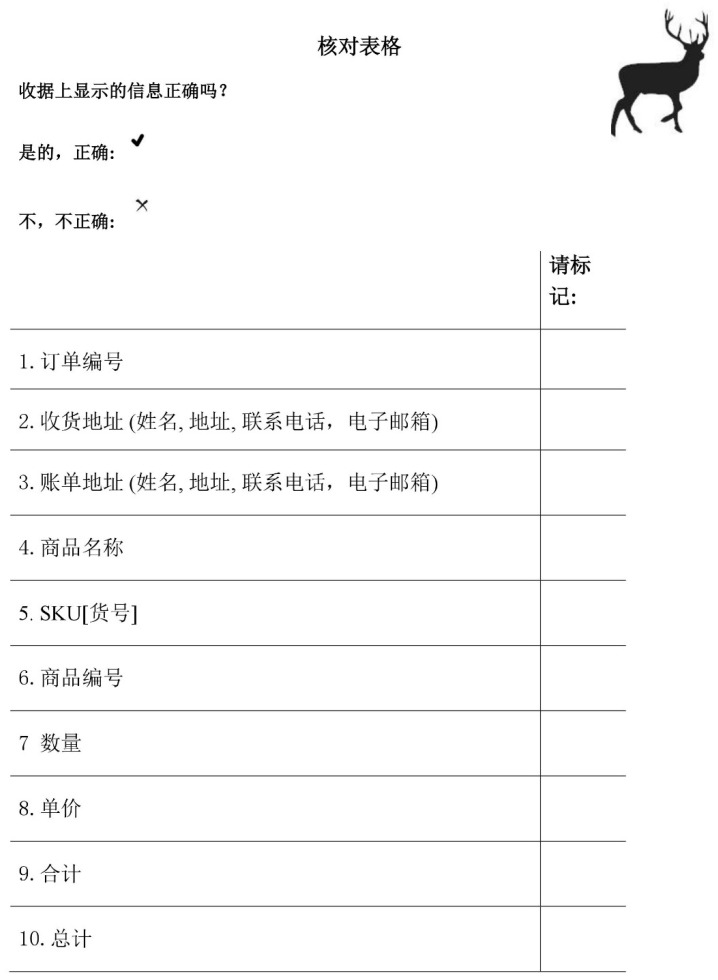
Study trial deer: Checksheet.

**Figure A16:**
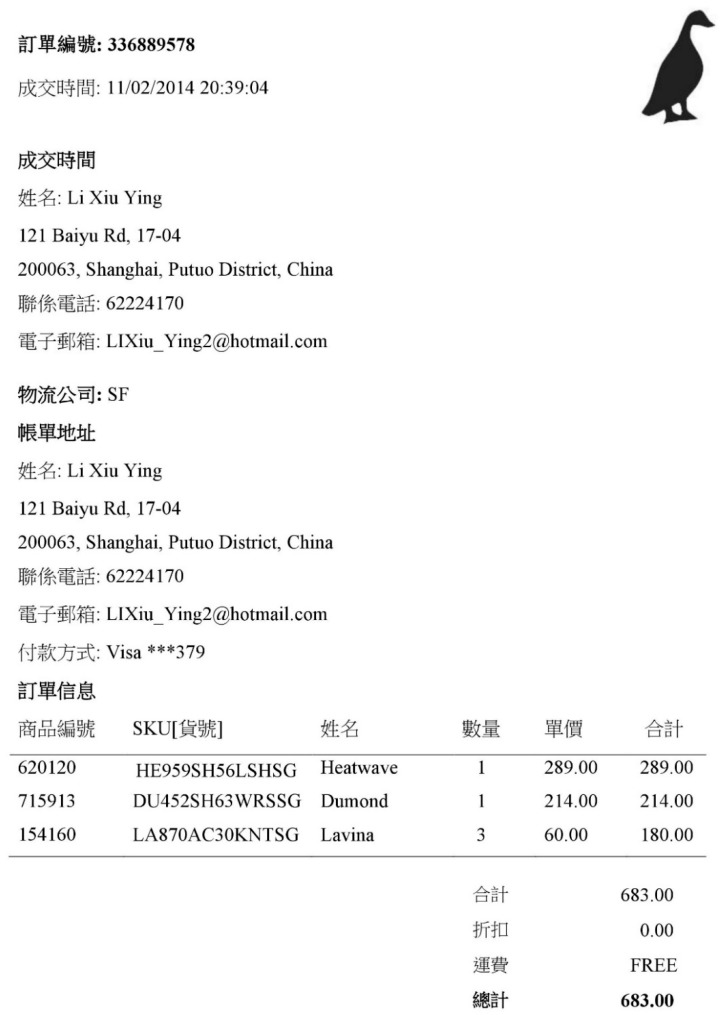
Study trial duck: Customer order.

**Figure A17:**
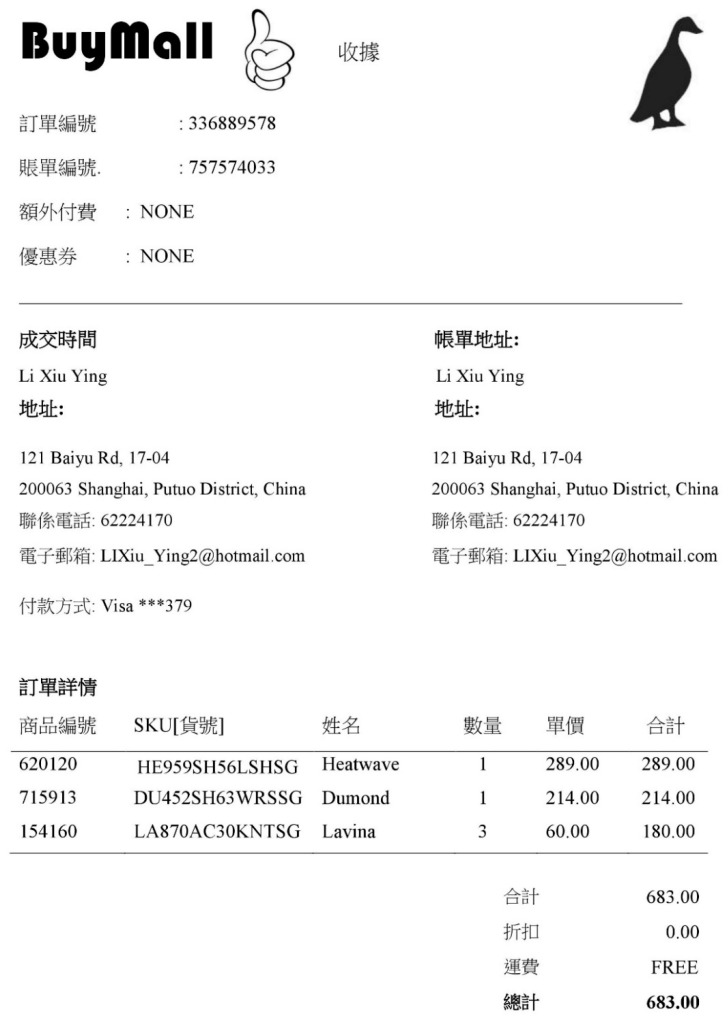
Study trial duck: Billing receipt.

**Figure A18:**
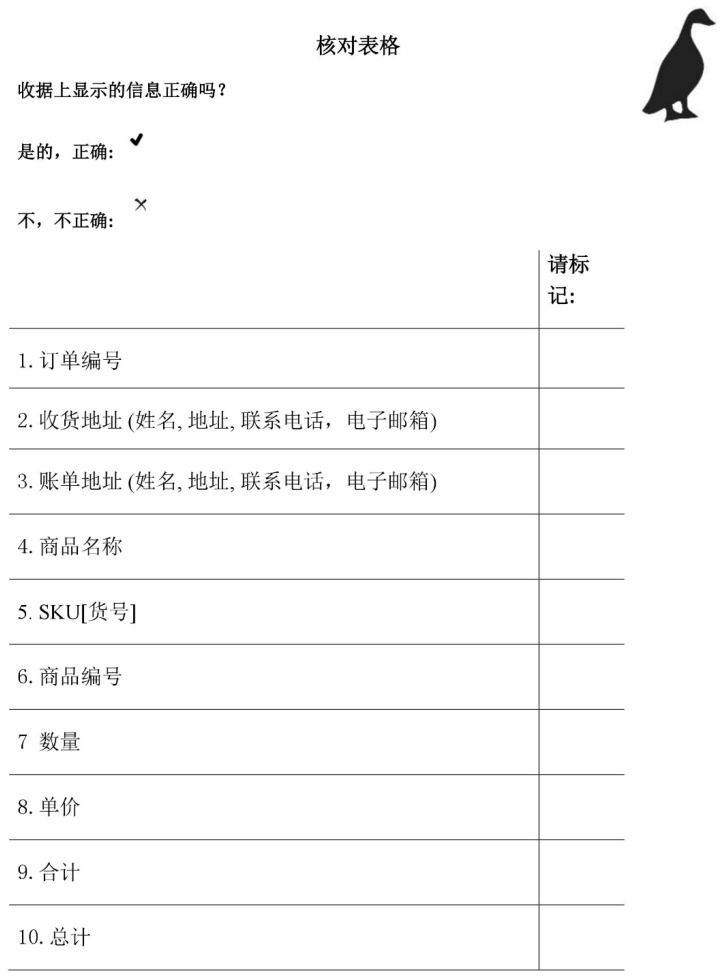
Study trial duck: Checksheet.

**Figure A19:**
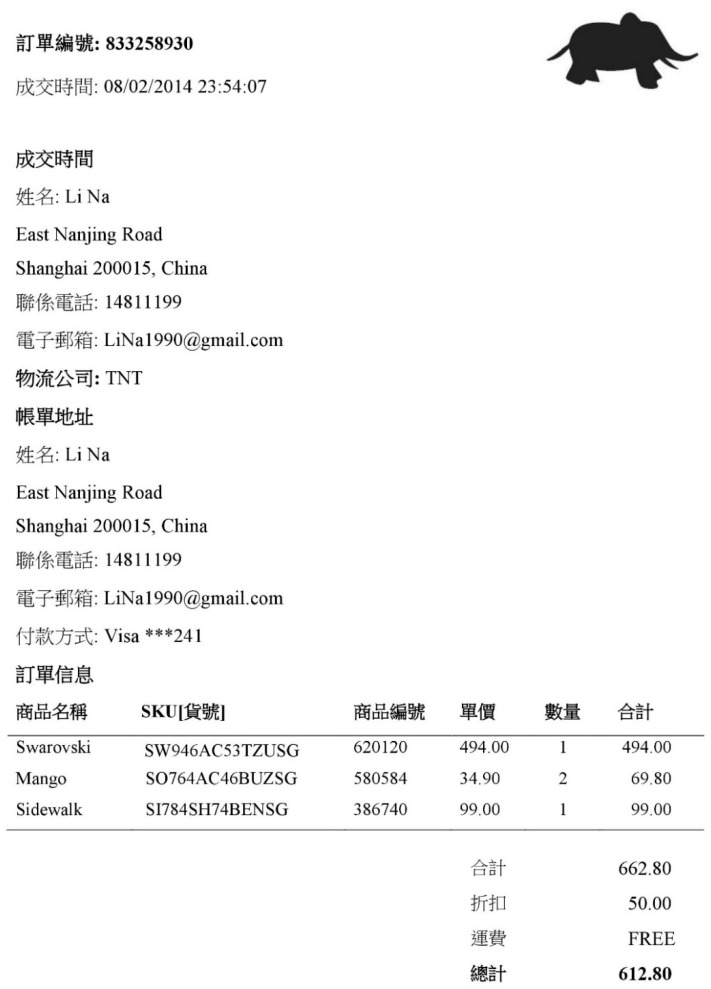
Study trial elephant: Customer order.

**Figure A20:**
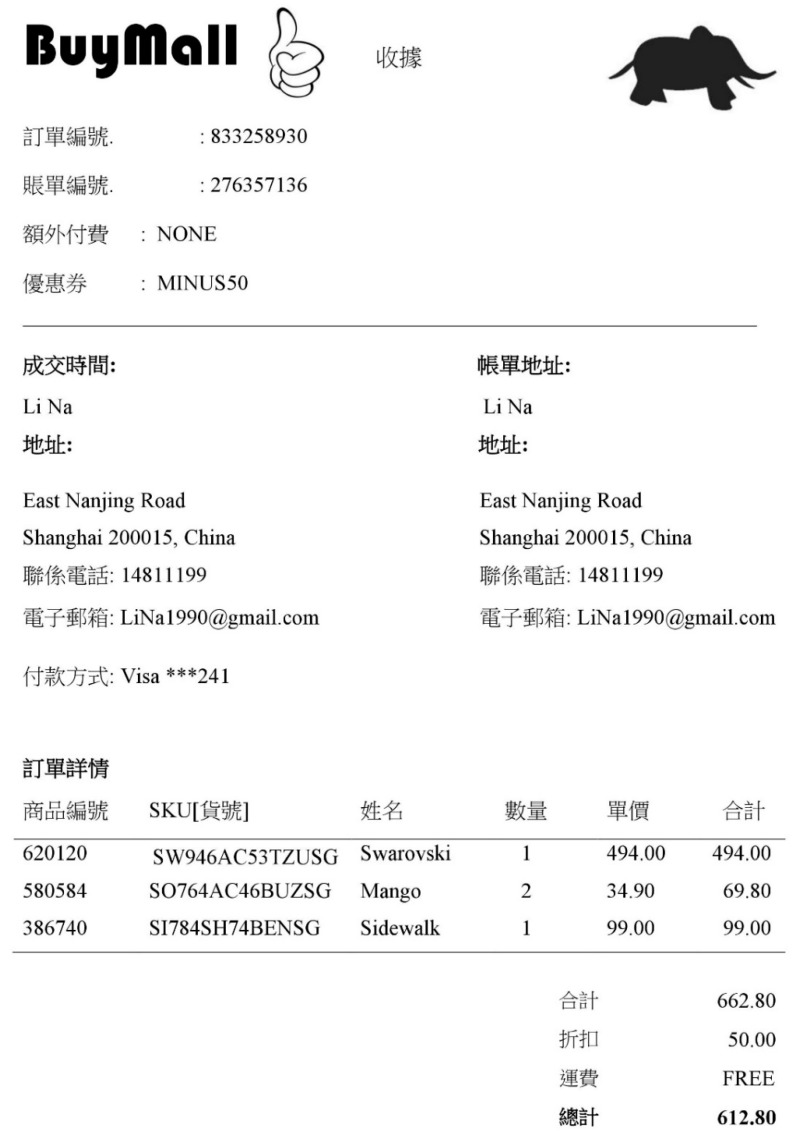
Study trial elephant: Billing receipt.

**Figure A21:**
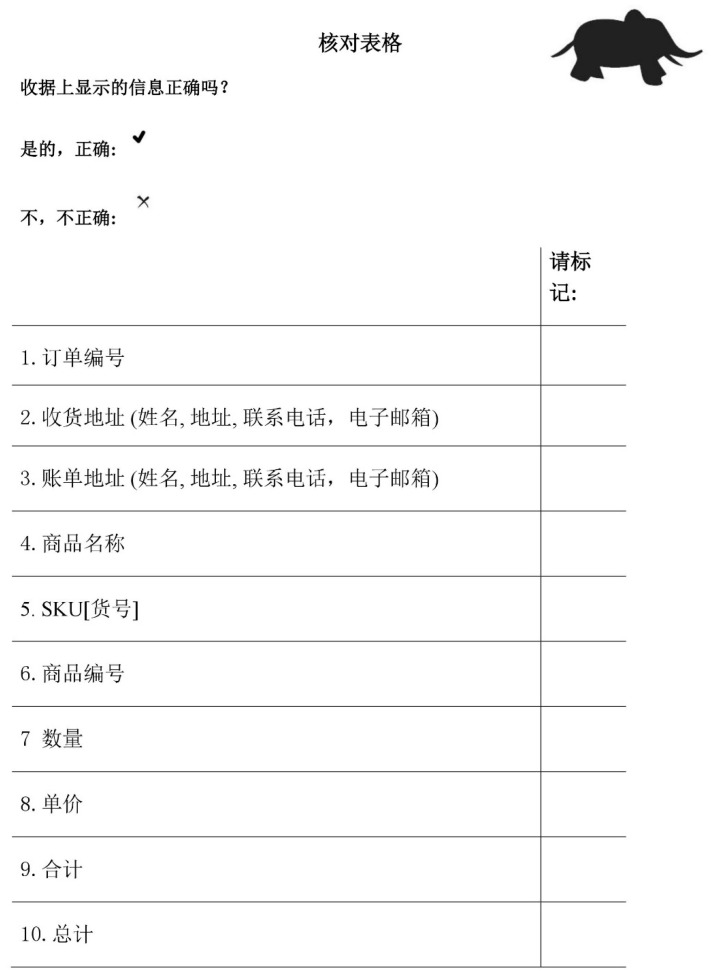
Study trial elephant: Checksheet.

**Figure A22:**
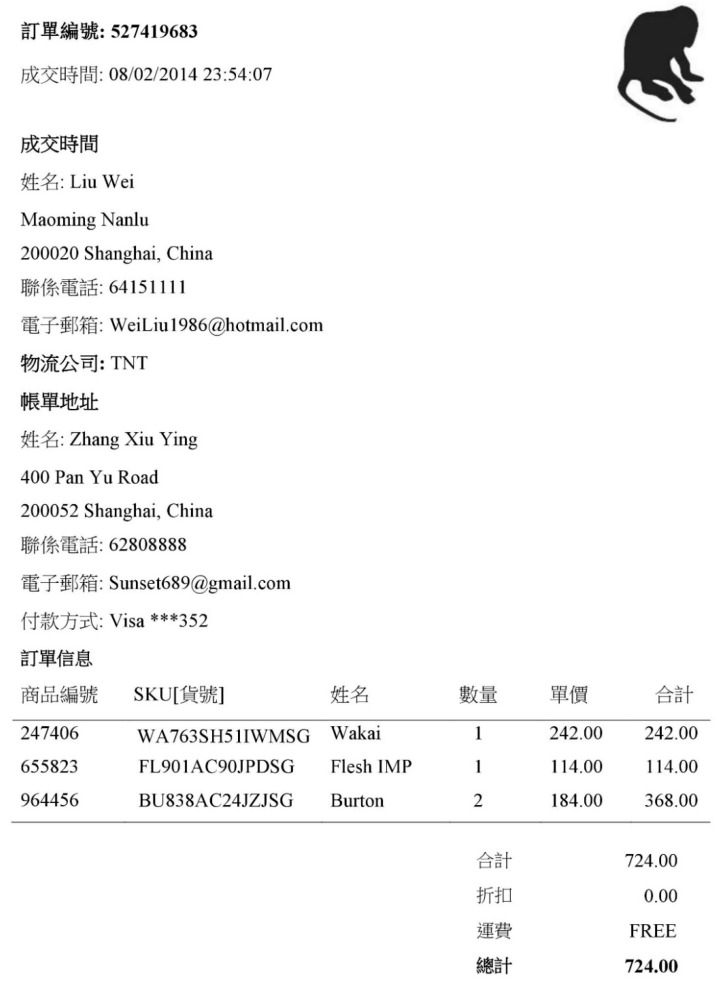
Study trial monkey: Customer order.

**Figure A23:**
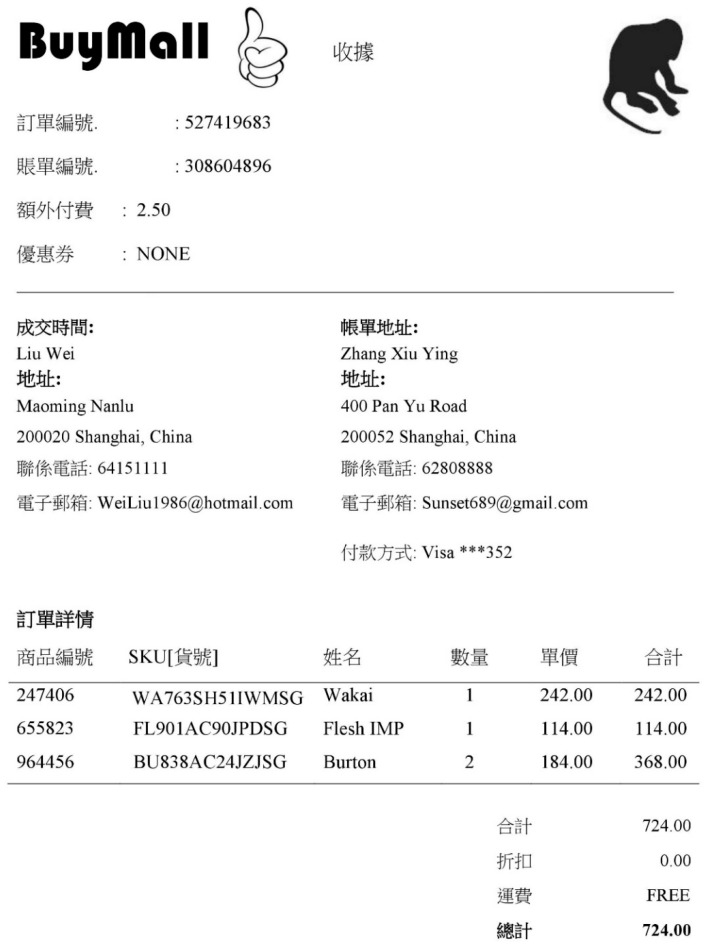
Study trial monkey: Billing receipt.

**Figure A24:**
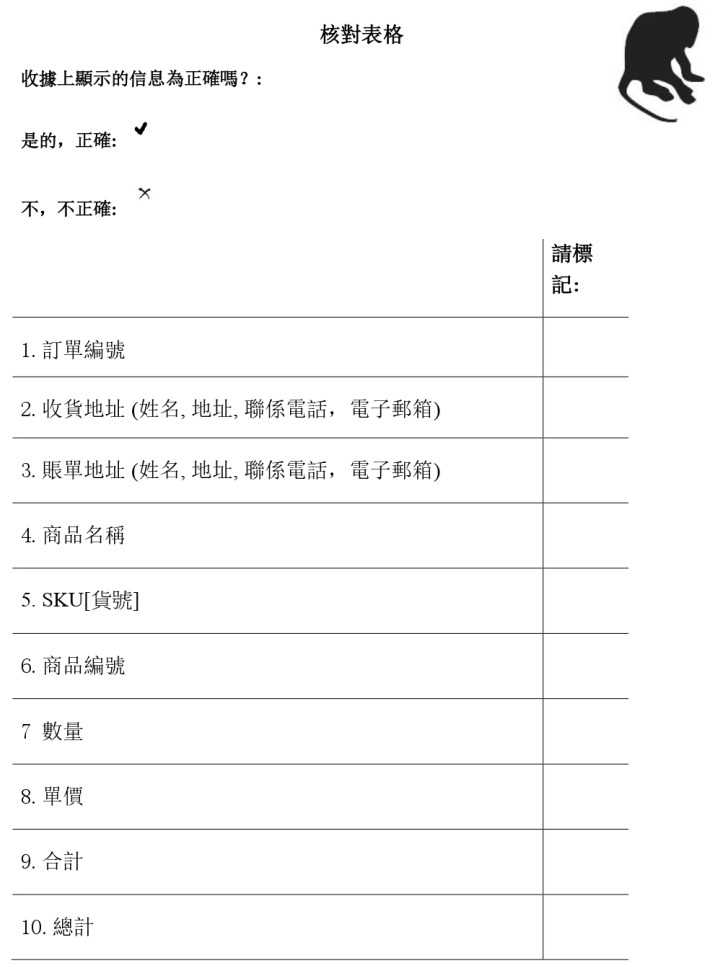
Study trial monkey: Checksheet.

**Figure A25:**
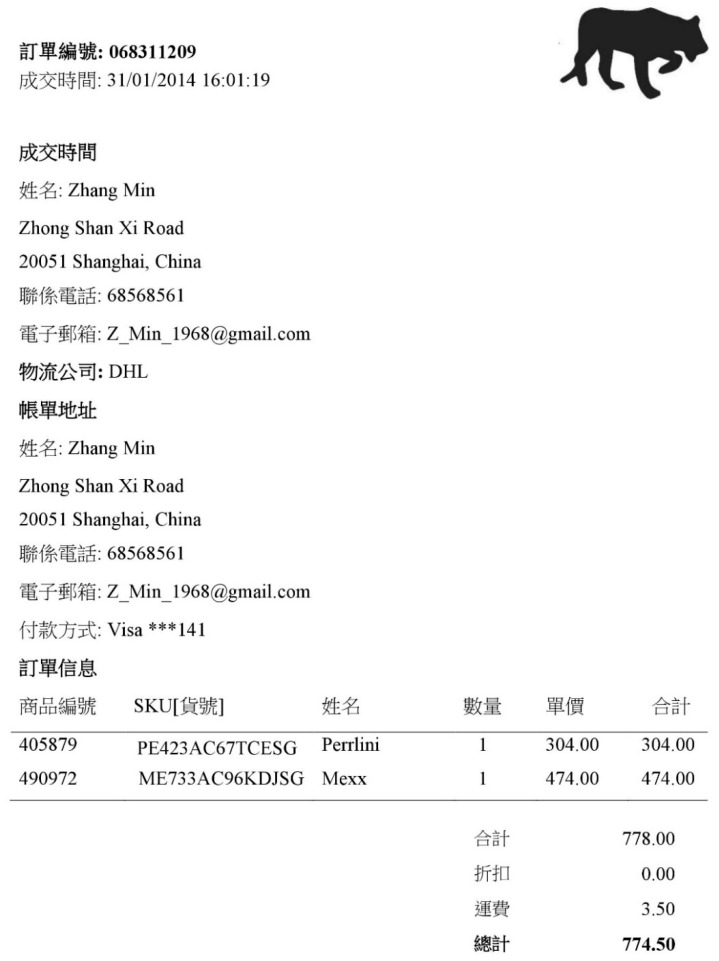
Study trial tiger: Customer order.

**Figure A26:**
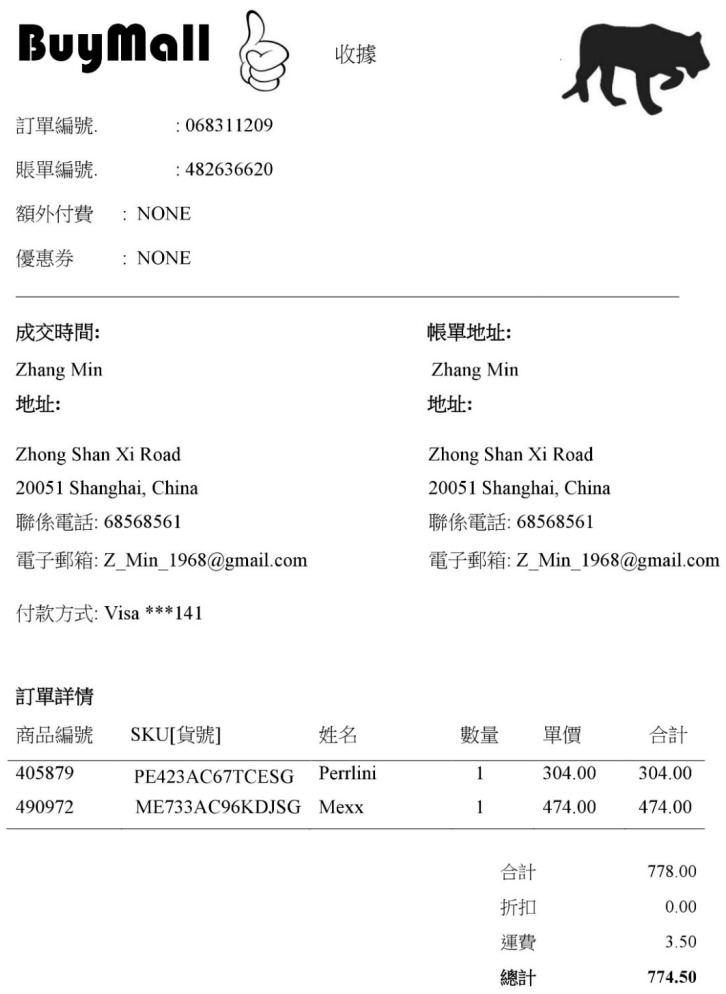
Study trial tiger: Billing receipt.

**Figure A27:**
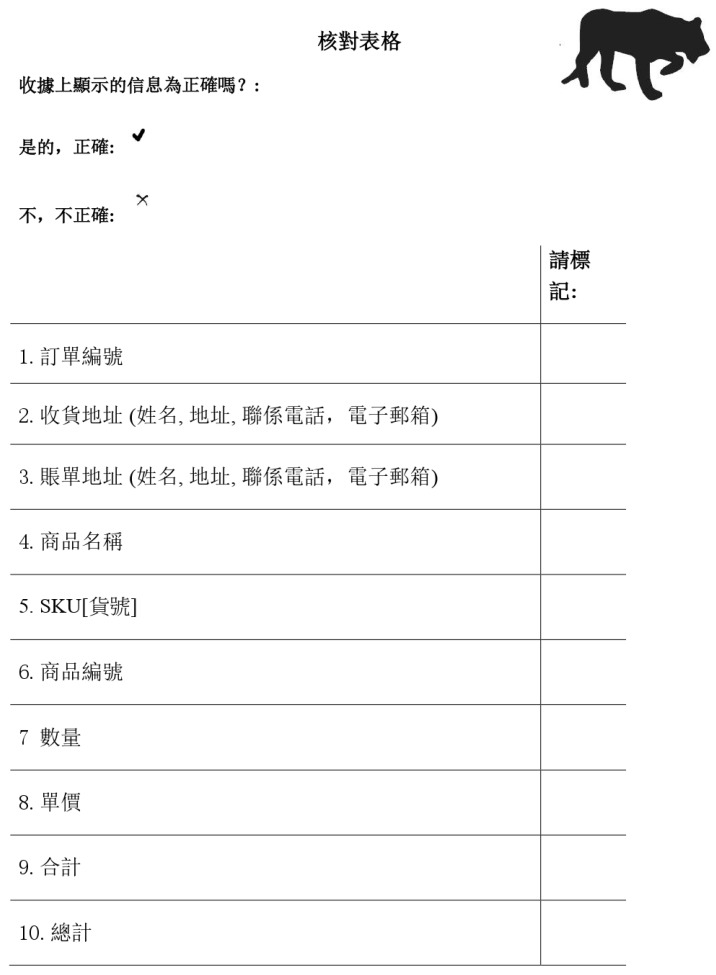
Study trial tiger: Checksheet.

**Figure A28:**
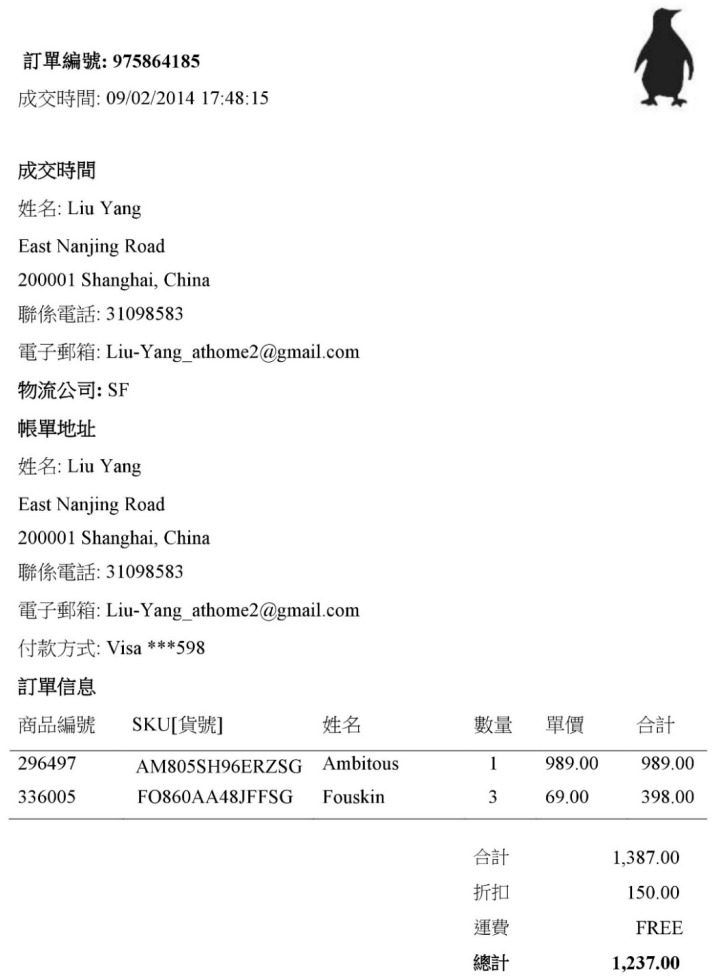
Study trial penguin: Customer order.

**Figure A29:**
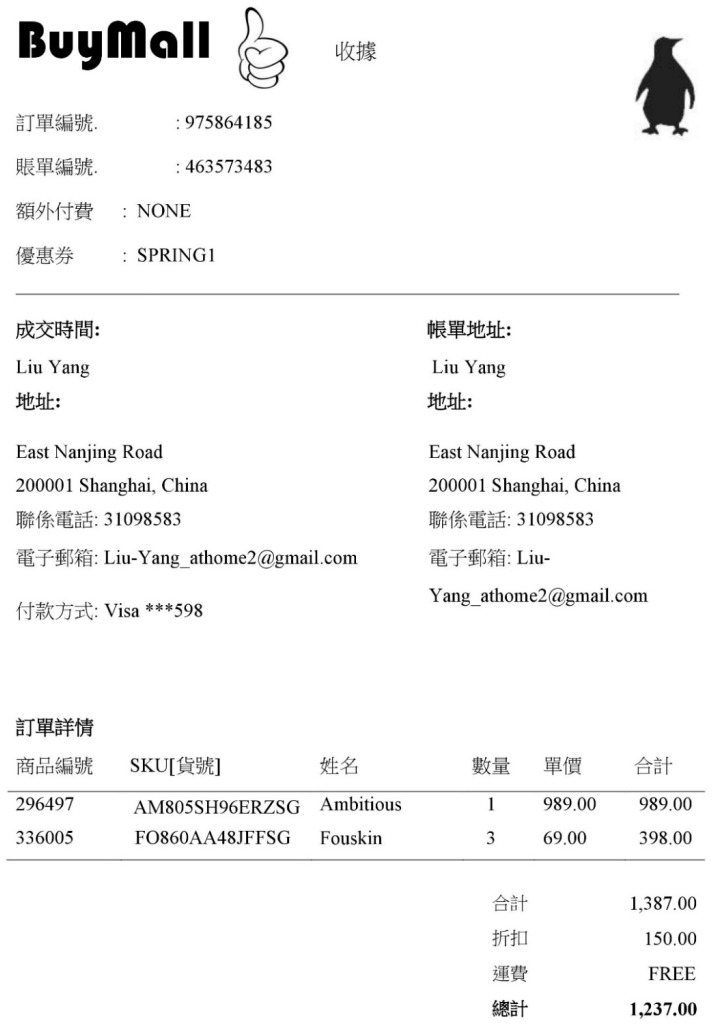
Study trial penguin: Billing receipt.

**Figure A30:**
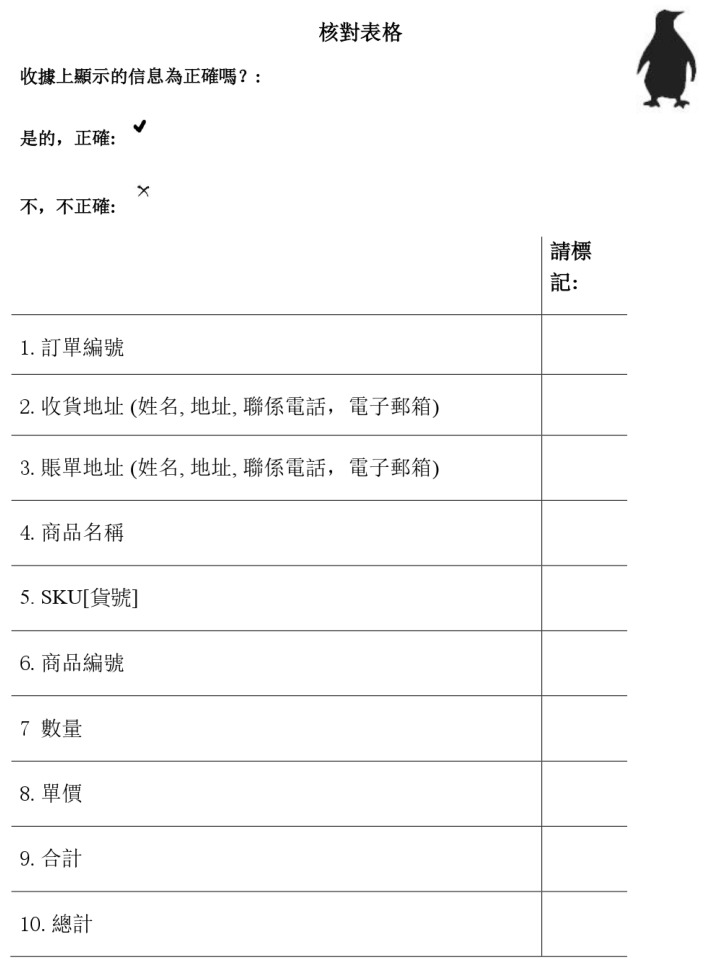
Study trial penguin: Checksheet.

**Figure A31:**
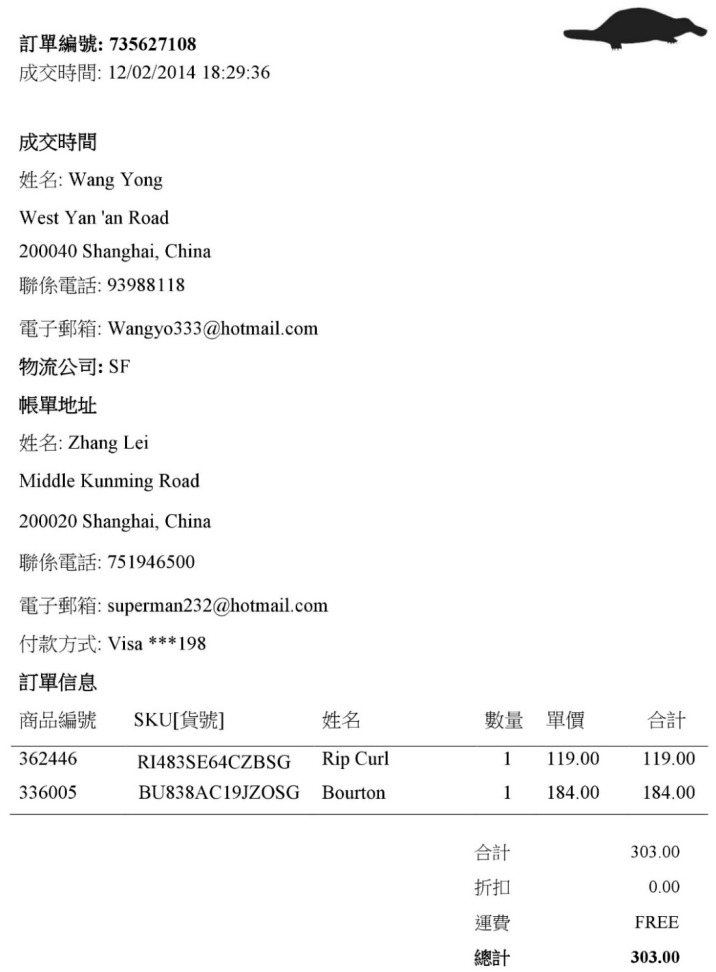
Study trial platypus: Customer order.

**Figure A32:**
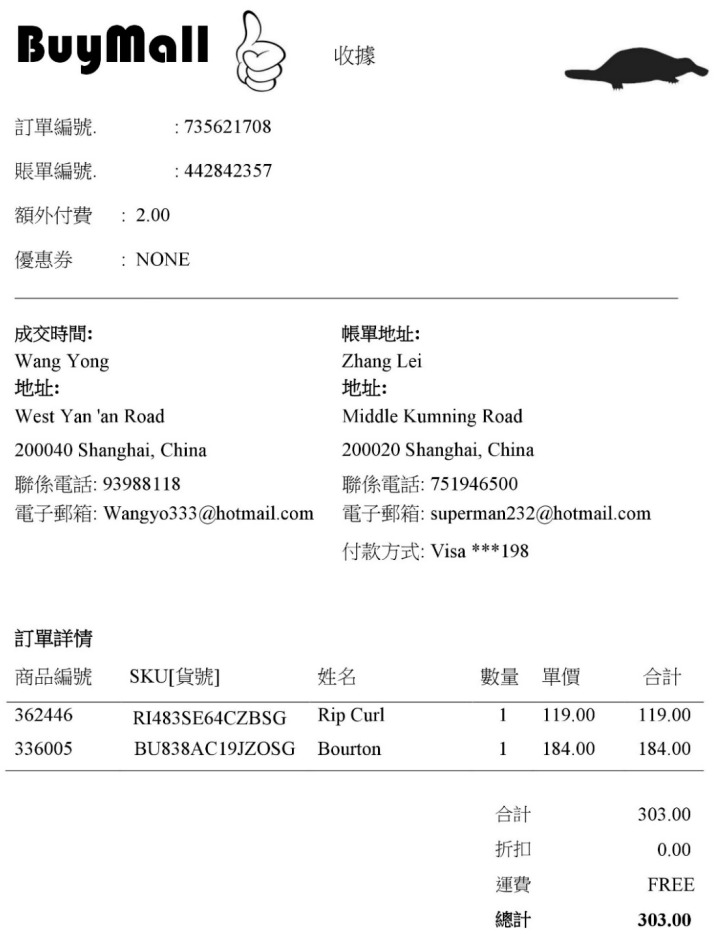
Study trial platypus: Billing receipt.

**Figure A33:**
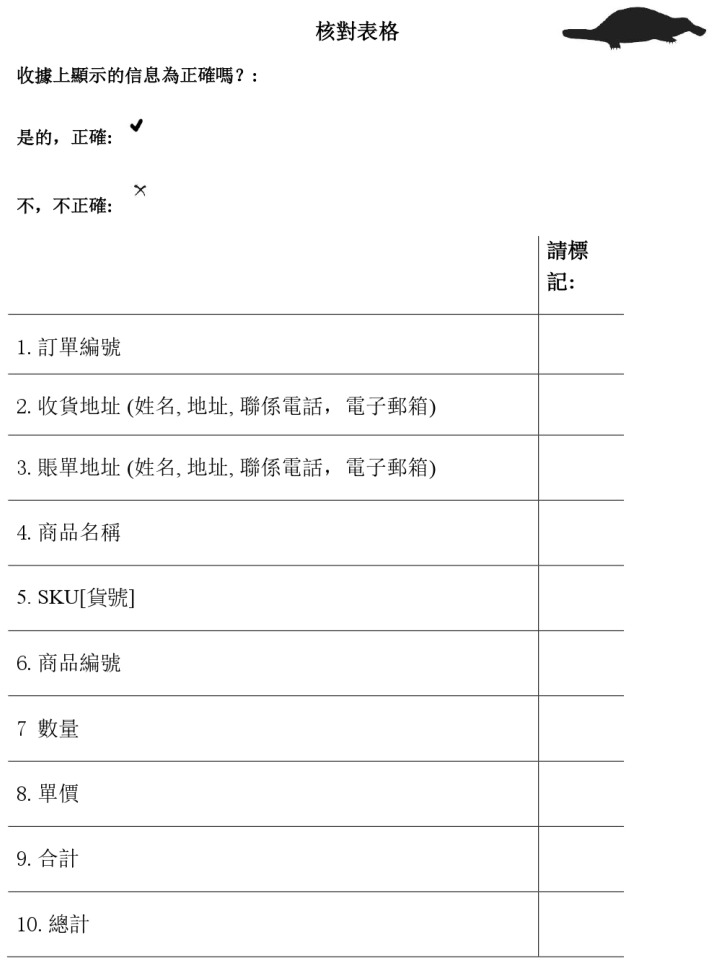
Study trial platypus: Checksheet.

**Figure A34:**
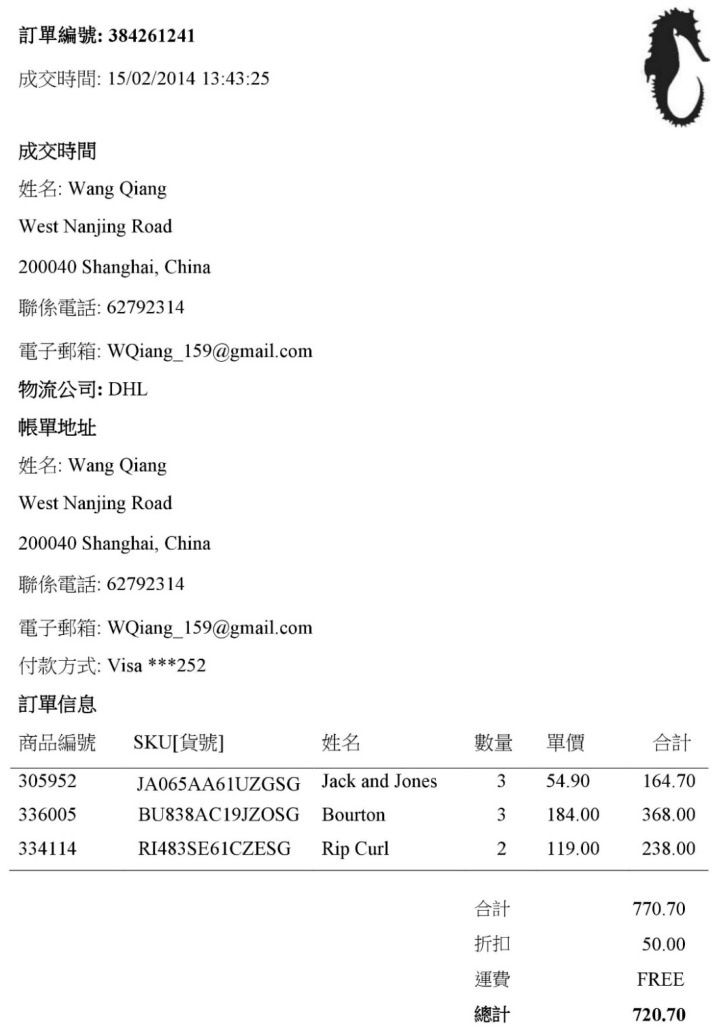
Study trial seahorse: Customer order.

**Figure A35:**
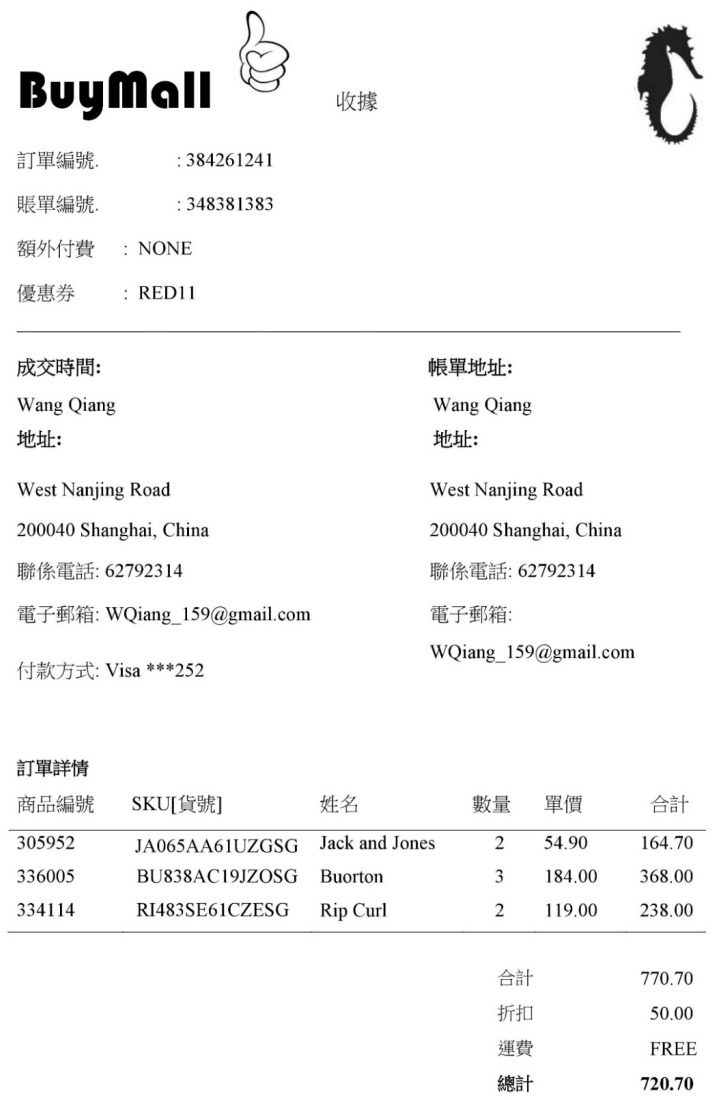
Study trial seahorse: Billing receipt.

**Figure A36:**
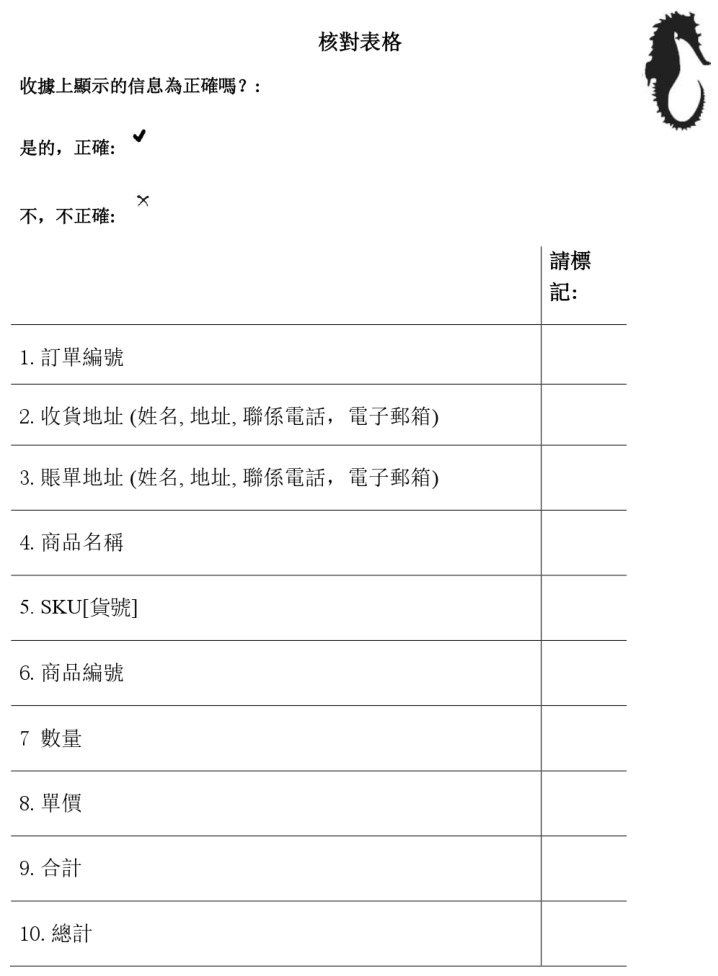
Study trial seahorse: Checksheet.

**Figure A37:**
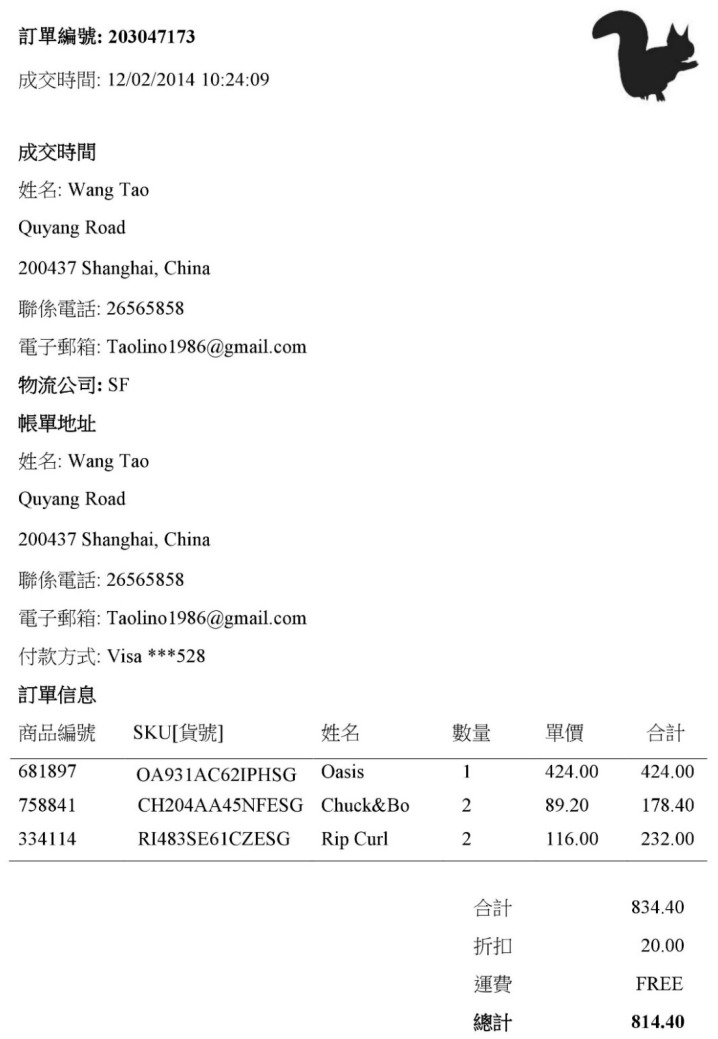
Study trial squirrel: Customer order.

**Figure A38:**
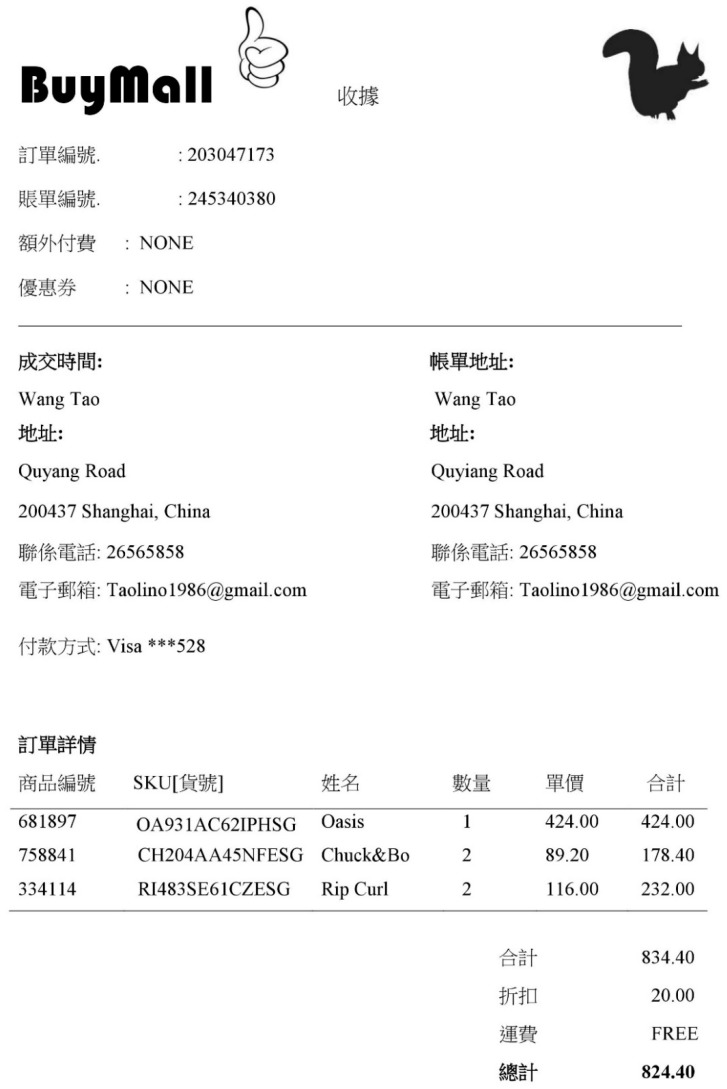
Study trial squirrel: Billing receipt.

**Figure A39:**
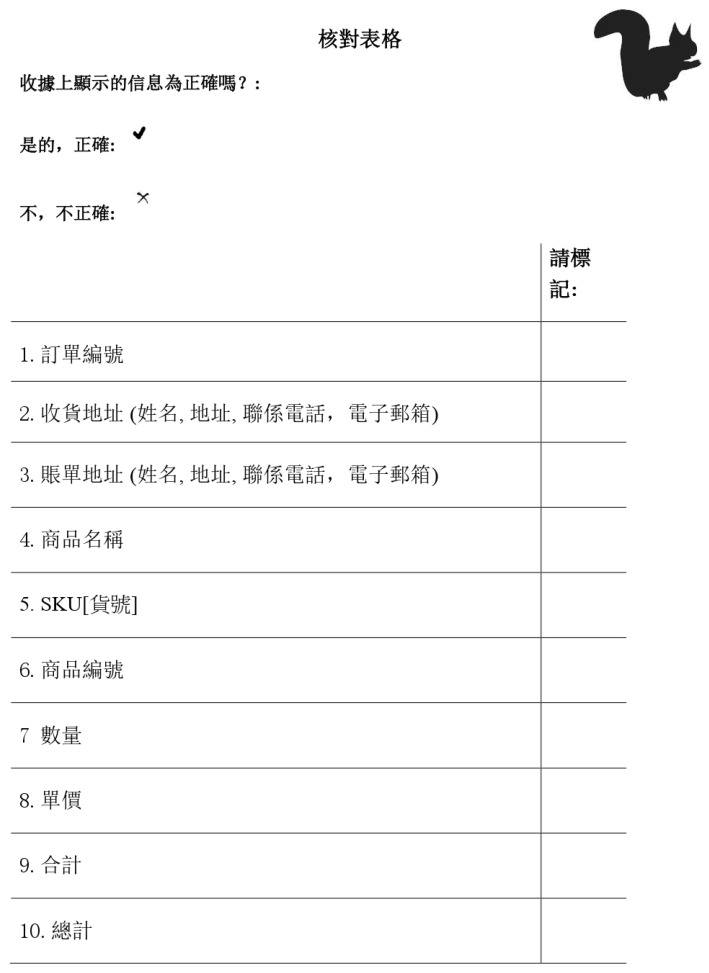
Study trial squirrel: Checksheet.

**Figure A40:**
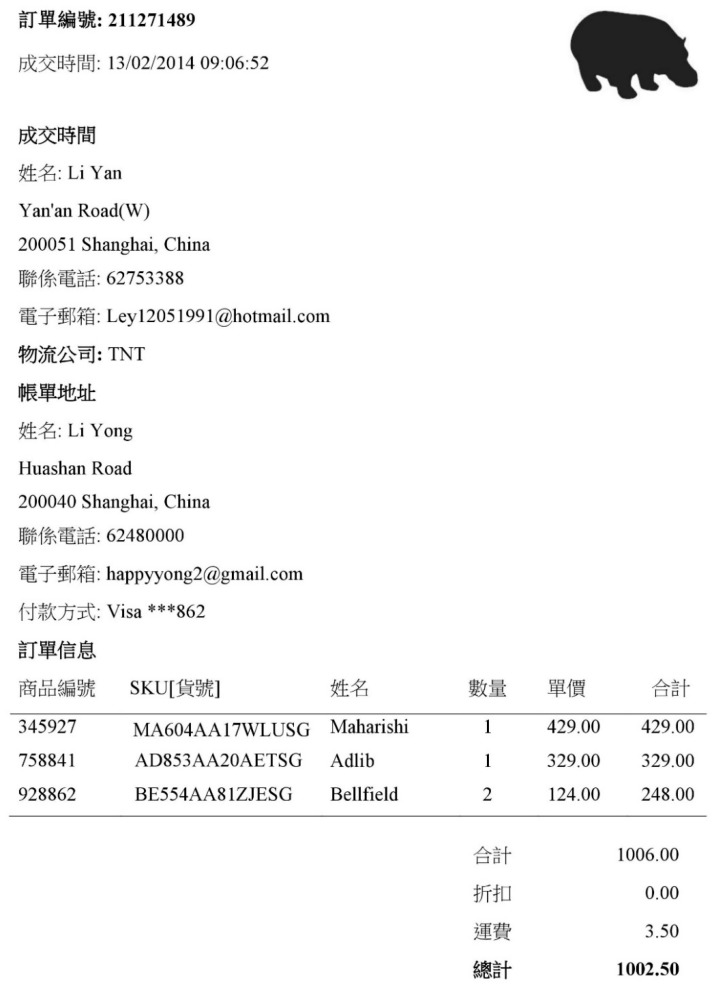
Study trial hippo: Customer order.

**Figure A41:**
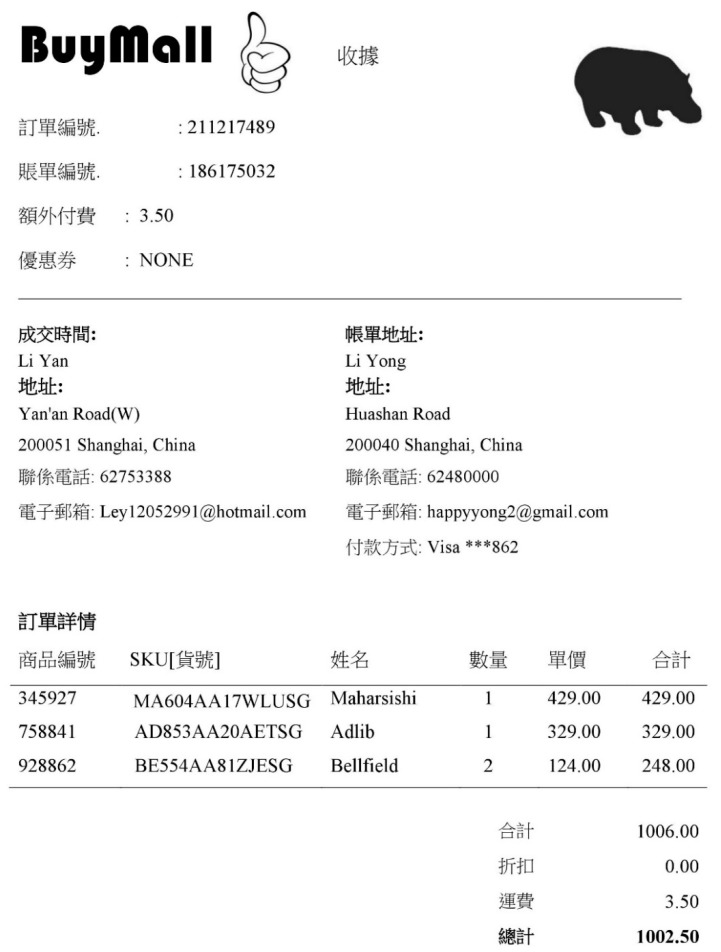
Study trial hippo: Billing receipt.

**Figure A42:**
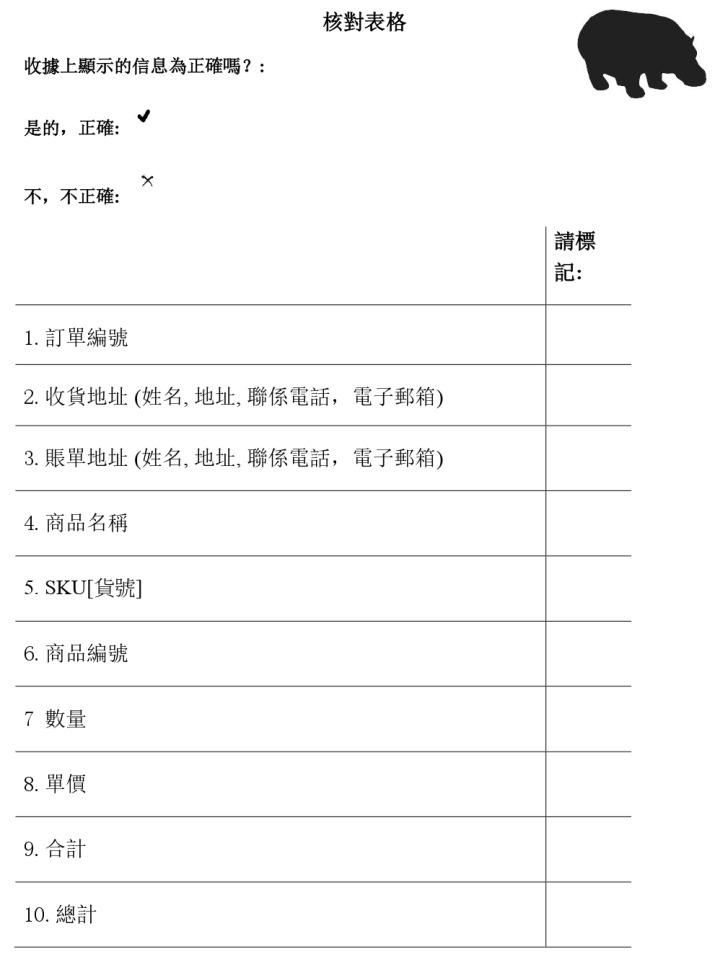
Study trial hippo: Checksheet.

## Appendix A

### Error Detection Task

In the following, all trials and checksheets from the error detection task are being displayed. The first two trials (marked with rabbit and cat symbols) are practice trials, the remaining twelve trials are study trials. Animal symbols indicate which trials correspond to each other, i.e., needed to be compared.

## Appendix B

### Illustration of the Procedure of the Error Detection Task

Participants were instructed to complete the following steps in the error detection task:

Step 1: Participants were presented with three sheets of paper in front of them: One) a customer order, two) a billing receipt and three) a checksheet. To prevent confusion, corresponding papers were easily identifiable by the same animal symbols in the top right corner.

Step 2: Participants were instructed to go through 10 items listed on the checksheet and identify for each whether it was correct or incorrect.

Step 3: Participants had to locate each item listed on the checksheet on both the customer order and the billing receipt to check whether it was correct. For example, the first item on the checksheet that needed to be located was the order number.

Step 4: Participants indicated whether the items were correct (i.e., the same on both the customer order and billing address) or whether there was an error on the checksheet. If they decided that the item was correct, they were asked to put a tick behind the respective category. If participants thought they had spotted an error, they were asked to put an “X” behind the respective category on the checksheet.
